# Bis(dicarbollide) Complexes of Transition Metals as a Platform for Molecular Switches. Study of Complexation of 8,8′-Bis(methylsulfanyl) Derivatives of Cobalt and Iron Bis(dicarbollides)

**DOI:** 10.3390/molecules25235745

**Published:** 2020-12-05

**Authors:** Sergey A. Anufriev, Sergey V. Timofeev, Alexei A. Anisimov, Kyrill Yu. Suponitsky, Igor B. Sivaev

**Affiliations:** 1 A.N. Nesmeyanov Institute of Organoelement Compounds, Russian Academy of Sciences, 28 Vavilov Str., 119991 Moscow, Russia; trueman476@mail.ru (S.A.A.); timofeev@ineos.ac.ru (S.V.T.); anisimov.alex.a@gmail.com (A.A.A.); kirshik@yahoo.com (K.Y.S.); 2 Higher Chemical College at the Russian Academy of Sciences, D.I. Mendeleev Russian Chemical Technological University, 9 Miusskaya Sq., 125047 Moscow, Russia; 3 Basic Department of Chemistry of Innovative Materials and Technologies, G.V. Plekhanov Russian University of Economics, 36 Stremyannyi Line, 117997 Moscow, Russia

**Keywords:** molecular switches, cobalt bis(dicarbollide), iron bis(dicarbollide), methylsulfanyl derivatives, complexation, X-ray diffraction

## Abstract

Complexation of the 8,8′-bis(methylsulfanyl) derivatives of cobalt and iron bis(dicarbollides) [8,8′-(MeS)_2_-3,3′-M(1,2-C_2_B_9_H_10_)_2_]^−^ (M = Co, Fe) with copper, silver, palladium and rhodium leads to the formation of the corresponding chelate complexes, which is accompanied by a transition from the *transoid* to the *cisoid* conformation of the bis(dicarbollide) complex. This transition is reversible and can be used in design of coordination-driven molecular switches based on transition metal bis(dicarbollide) complexes. The solid-state structures of {(Ph_3_P)ClPd[8,8′- (MeS)_2_-3,3′-Co(1,2-C_2_B_9_H_10_)_2_-κ^2^-S,S′]} and {(COD)Rh[8,8′-(MeS)_2_-3,3′-Co(1,2-C_2_B_9_H_10_)_2_-κ^2^-S,S′]} were determined by single crystal X-ray diffraction.

## 1. Introduction

The design of molecular machines—molecules or molecular complexes that are capable of performing quasi-mechanical movement to do useful work—is one of the most attractive and rapidly developing areas of modern chemistry [1,2,3,4]. The importance and topicality of this area was recognized by the 2016 Nobel Prize in Chemistry awarded to Jean-Pierre Sauvage, Bernard Feringa, and Fraser Stoddart “for the design and synthesis of molecular machines” [5,6,7]. Depending on the type of motion, all molecular machines can be divided into two main groups—performing linear or rotational motion. In turn, molecular machines that carry out rotational motion can also be divided into two types—molecular motors (rotors) and molecular switches [8]. Molecular switches are molecules or supramolecular complexes that can exist in two or more stable forms, differing in the mutual orientation of their components, and which, under the influence of external factors, can be converted from one form to another due to the rotation (rotation) of these components relative to each other. Currently, the most studied are molecular switches based on organic molecules, including photochromic molecular switches based on azobenzenes, stilbenes, dithienylethenes, spiropyrans, and spirooxazines, as well as switches based on mechanically interlocked molecular systems, rotoxanes and catenanes, in which the bistable states differ in the relative positions of the parts [9]. At the same time, despite the significant progress achieved in the synthesis and study of such molecules, there are some problems caused by relatively low stability of many organic materials to atmospheric oxygen and moisture, that stimulates the search for new types of compounds that can be used as a structure-forming component in the design of effective molecular switches. Therefore, there is growing interest in molecular switches and other molecular electronics devices based on transition metal complexes [10,11,12,13,14]. However, as in the case of organic molecular machines, devices based on transition metal complexes often have low stability. Therefore, the choice of a stable organometallic module is of particular importance for the design of molecular electronics devices. Examples of such organometallic modules are ferrocene and bis(dicarbollide) complexes of iron group metals [15]. The last ones demonstrate extraordinary chemical stability and practically unlimited possibilities for chemical modification by directed replacement of hydrogen atoms with various atoms and functional groups [16,17,18].

Recently we proposed the 8,8′-bis(methylsulfanyl) derivatives of cobalt, iron and nickel bis(dicarbollides) as organometallic modules for complexation driven molecular switches. These derivatives adopt the *transoid* configuration with the dicarbollide ligands rotated 180° relative to each other due to the formation of weak intramolecular hydrogen bonds CH…S(Me) between the ligands [19,20,21]. It is assumed that, in the presence of external complexing metals, these weak intramolecular bonds should break with the formation of stronger donor-acceptor bonds S(Me)→M with the metal ion. If there are two free sites in the coordination sphere of the metal, it should lead to the formation of a chelate complex with a change in the conformation of cobalt bis(dicarbollide) from the *transoid* to the *cisoid* one. In turn, since thioesters are weak ligands, the addition of a stronger ligand should lead to release of the 8,8′-bis(methylsulfanyl) bid(dicarbollides) from the coordination sphere of the metal with their reverse transition from the *cisoid* to the *transoid* conformation.

In this contribution we report the complexation of cobalt and iron bis(dicarbollides) with some transition metals, including copper, silver, palladium and rhodium. 

## 2. Results and Discussion

Our previous attempt to obtain a chelate complex of 8,8′-bis(methylsulfanyl) cobalt bis(dicarbollide) [8,8′-(MeS)_2_-3,3′-Co(1,2-C_2_B_9_H_10_)_2_]^−^ and tetracarbonyl tungsten was unsuccessful due to the carbonyl rearrangement and led to the formation of stable pentacarbonyl tungsten complexes {(CO)_5_W [8,8′-(MeS)_2_-3,3′-Co(1,2-C_2_B_9_H_10_)_2_-κ-*S*]}^−^ and {[(CO)_5_W]_2_[8,8′-(MeS)_2_-3,3′-Co(1,2-C_2_B_9_H_10_)_2_-κ^2^-*S,S′*]}^−^ separated as the tetrabutylammonium salt [22]. However, this study gave us a proper understanding of how the coordination is reflected in the NMR spectra of the complexes (See Supplementary Materials NMR spectra of compounds **2**–**6** and **8**). In addition to a downfield shift of the signal of the methylsulfanyl group in the ^1^H and ^13^C NMR spectra, the value of which to some extent reflects the strength of the sulfur-metal bond, the most characteristic is a strong narrowing of the signal of the substituted boron atom in the ^11^B NMR spectra together with the broadening of the signals of unsubstituted boron atoms. The last one makes it possible to observe the metal coordination in the reaction mixture, while the shift of the SMe group signal in the case of weakly bound complexes can be insignificant [23,24,25,26], and the signal itself can be “masked” by the signals of the solvent, ligand, or accidental water.

In this study, the singly charged diamagnetic cations of copper(I) and silver(I) were chosen to study the complexation of [8,8′-(MeS)_2_-3,3′-Co(1,2-C_2_B_9_H_10_)_2_]^−^ in order to facilitate the isolation and purification of the resulting complexes, as well as their characterization using NMR spectroscopy.

**Scheme 1:**
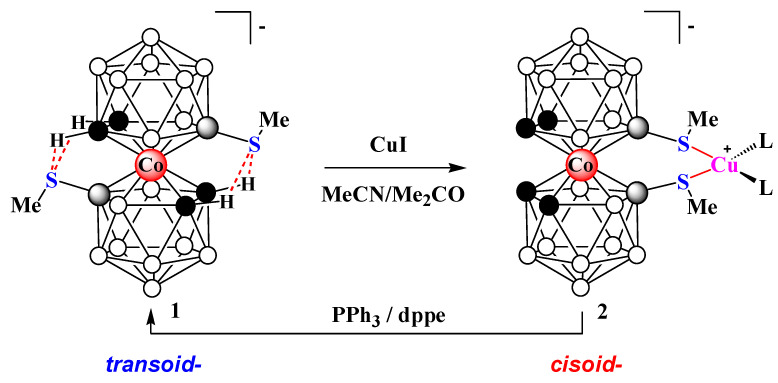
Complexation of [8,8′-(MeS)_2_-3,3′-Co(1,2-C_2_B_9_H_10_)_2_]^−^ with copper.

The reaction of (Me_3_NH)[8,8′-(MeS)_2_-3,3′-Co(1,2-C_2_B_9_H_10_)_2_] ((Me_3_NH)[1]) with CuI in acetonitrile leads to the formation of a neutral purple complex {L_2_Cu[8,8′-(MeS)_2_-3,3′-Co(1,2- C_2_B_9_H_10_)_2_-κ^2^-S,S′]} (**2**) which is accompanied by the transformation of the *transoid* conformation of cobalt bis(dicarbollide) **1**, stabilized by intramolecular hydrogen CH…S(Me) bonds [19], to the *cisoid* one, stabilized by dative bonds to the metal (Scheme 1). The complexation results in the downfield shift of the signal of the MeS-substituted boron atoms in the ^11^B NMR spectrum from 11.3 to 15.8 ppm, while the signal of the MeS groups in the ^1^H NMR spectrum demonstrate the downfield shift from 1.87 to 2.21 ppm. Despite the fact that the coordination of copper with anion **1** is retained during chromatographic purification, our numerous attempts to obtain crystals of complex **2** suitable for X-ray diffraction study were unsuccessful, which may be due to both weak coordination of solvent molecules (acetone or acetonitrile) as well as due to the possibility of the copper coordination by B-H groups of the anion, similar to that previously found in {(Ph_3_P)Cu[3,3′-Co(1,2-C_2_B_9_H_11_)_2_]} [27] and some other complexes with polyhedral boron hydride anions [28,29,30]. At the same time, attempts to replace the solvent molecules in the coordination sphere of copper with stronger ligands, such as 1,2-bis(diphenylphosphino)ethane or triphenylphosphine, lead to the destruction of complex **2** with the release of free thioether ligand **1** (Scheme 1).

**Scheme 2:**
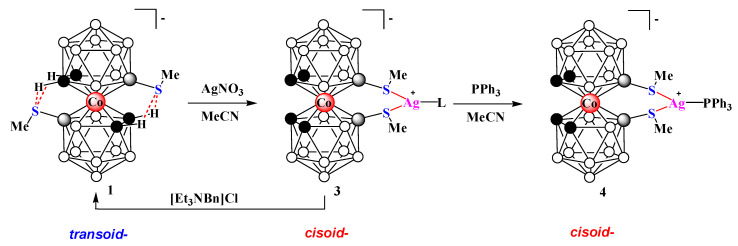
Complexation of [8,8′-(MeS)_2_-3,3′-Co(1,2-C_2_B_9_H_10_)_2_]^−^ with silver.

**Scheme 3:**
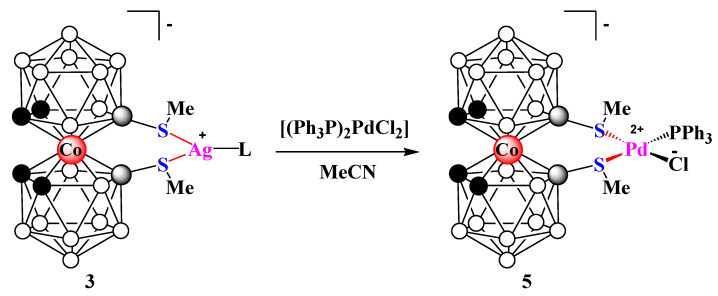
Reaction of {LAg[8,8′-(MeS)_2_-3,3′-Co(1,2-C_2_B_9_H_10_)_2_-κ^2^-S,S′]} with [(Ph_3_P)_2_PdCl_2_].

In a similar way, the reaction of ((Me_3_NH)[1]) with AgNO_3_ in acetonitrile leads to the corresponding silver complex {LAg[8,8′-(MeS)_2_-3,3′-Co(1,2-C_2_B_9_H_10_)_2_-κ^2^-S,S′]} (**3**) (Scheme 2). The complexation results in the downfield shift of signals of the substituted boron atoms and the MeS groups in the ^11^B NMR and ^1^H spectra to 13.9 and 2.19 ppm, respectively. In the silver complex **3**, as in the case of the copper complex **2**, the coordination sphere of the metal can be completed by both solvent molecules and the BH groups of cobalt bis(dicarbollide) [31,32,33]. Silver can be easily removed from the complex **3** by the treatment with benzyltriethylammonium chloride, which leads to the reverse transformation of the *cisoid* conformation to the *transoid* one. In contrast to the complex **2**, the reaction of complex **3** with triphenylphosphine proceeds without breaking the silver-sulfur bonds resulting in {(Ph_3_P)Ag[8,8′-(MeS)_2_-3,3′-Co(1,2-C_2_B_9_H_10_)_2_-κ^2^-S,S′]} (**4**) (Scheme 3). The complexation with such a strong ligand as triphenylphosphine leads to some weakening of the bond of silver with the methylsulfide ligand, which is reflected in the high field shift of signals of the substituted boron atoms and the MeS groups in the ^11^B NMR and ^1^H spectra to 12.8 and 1.97 ppm, respectively. It should be noted that, unlike the copper complex **2**, the silver complexes **3** and **4** are destroyed on a chromatographic column.

The reaction of **3** with bis(triphenylphosphine) palladium dichloride [(Ph_3_P)_2_PdCl_2_] in acetonitrile results in the chloride abstraction with the formation of the asymmetrical palladium complex {(Ph_3_P)ClPd[8,8′-(MeS)_2_-3,3′-Co(1,2-C_2_B_9_H_10_)_2_-κ^2^-S,S′]} (**5**) (Scheme 3). It should be noted that the direct complexation reaction of **1** with [(Ph_3_P)_2_PdCl_2_] does not proceed, and the formation of complex **5** occurs only upon the addition of silver nitrate. The signals of two non-equivalent SMe groups in the ^1^H NMR spectrum of **5** is located at 2.18 and 1.91 ppm (See ESI). The asymmetry of the palladium coordination environment is reflected also in the ^11^B NMR spectrum of **5** which demonstrates two narrow singlets at 15.1 and 11.2 ppm corresponding two substituted boron atoms with different ligands in the *trans*-position.

**Figure 1:**
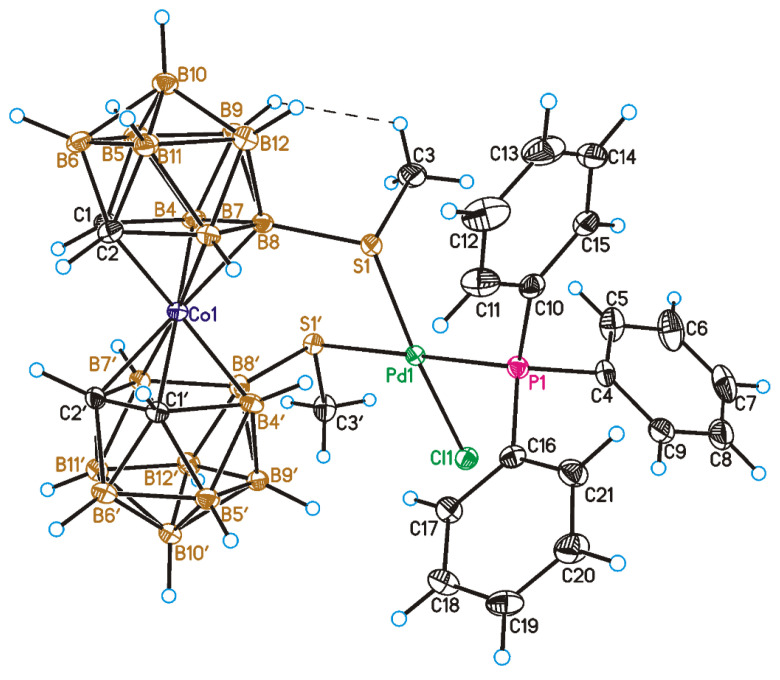
General view of complex **5** showing atomic numbering. Thermal ellipsoids are drawn at 50% probability level. Noncovalent C3-H3B…H9-B9 contact is shown by dashed line.

The solid-state structure of complex **5** was determined by single crystal X-ray diffraction. An asymmetric unit cell of complex **5** contains one molecule (Figure 1). The dicarbollide ligands in **5** adopt the *cisoid* conformation with pseudotorsion angle B8…Centroid(C1-C2-B7-B8-B4)…Centroid(C1′-C2′-B7′-B8′-B4′)…B8′ equal to −37.7(4)°. The palladium atom in **5** has slightly distorted square-planar geometry; the S-Pd-S angle is 86.50(3)° and the Pd-S bond lengths are 2.3020(10) and 2.3733(10) Å for the sulfur atoms in *trans-*position to the chloride and phosphine ligands, respectively. The B-S bond lengths are also some different (1.914(4) and 1.898(4) Å for B8-S1 and B8′-S1′, respectively) reflecting different bond strength of the methylsulfanyl groups with the palladium atom. There is shortened contact C3-H3B…H9-B9 between one methyl group and the dicarbollide ligand that is equal to 2.34 Å.

The difference in the binding strength of the palladium atom with the MeS groups, which is reflected in different Pd-S bond lengths and positions of the signals of the substituted boron atoms and the MeS groups in the NMR spectra, is in good agreement with the greater *trans* influence of the PPh_3_ ligand [34,35].

**Scheme 4:**
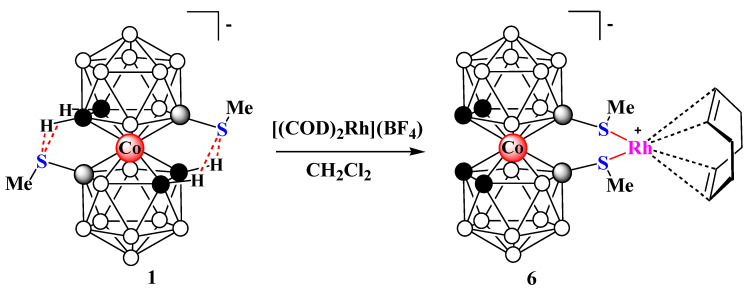
Complexation of [8,8′-(MeS)_2_-3,3′-Co(1,2-C_2_B_9_H_10_)_2_]^−^ with rhodium.

The symmetrical rhodium complex {(COD)Rh[8,8′-(MeS)_2_-3,3′-Co(1,2-C_2_B_9_H_10_)_2_-κ^2^-S,S′]} (**6**) was synthesized by the reaction of Cs[1] with bis(1,5-cyclooctadiene)rhodium(I) tetrafluoroborate [(COD)_2_Rh](BF_4_) in dichloromethane (Scheme 4). The complexation results in the downfield shift of signals of the substituted boron atoms and the MeS groups in the ^11^B NMR and ^1^H spectra to 13.4 and 2.07 ppm, respectively.

**Figure 2:**
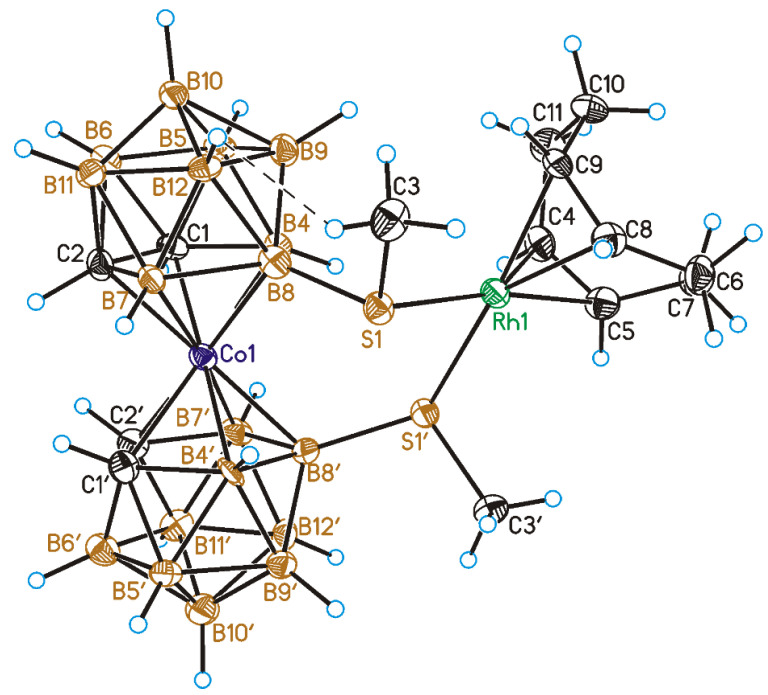
General view of complex **6** showing atomic numbering. Thermal ellipsoids are drawn at 50% probability level. Noncovalent C3-H3A…H12-B12 contact is shown by dashed line.

The solid-state structure of complex **6** was determined by single crystal X-ray diffraction (Figure 2). Complex **6** crystallizes in the form of solvate with chloroform. An asymmetric unit cell contains two independent Co-Rh-complexes and two chloroform molecules. The dicarbollide ligands in **6** adopt the *cisoid* conformation with the pseudotorsion angles B8…Centroid(C1-C2-B7-B8-B4)… Centroid(C1′-C2′-B7′-B8′-B4′)…B8′ equal to −38.1(7)° and 39.4(7)° for two independent molecules. The short contacts C3-H3A…H12-B12 for two independent molecules correspond to 2.39 and 2.37 Å.

**Scheme 5:**
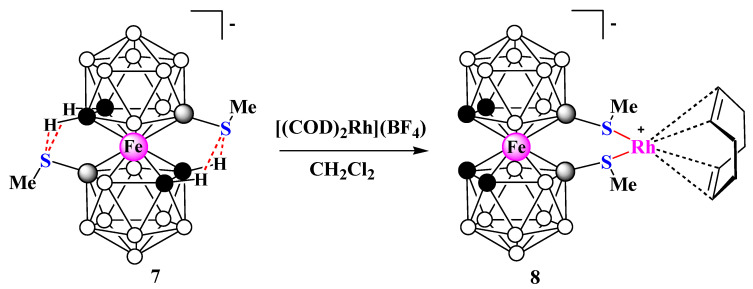
Complexation of [8,8′-(MeS)_2_-3,3′-Fe(1,2-C_2_B_9_H_10_)_2_]^−^ with rhodium.

The similar paramagnetic complex {(COD)Rh[8,8′-(MeS)_2_-3,3′-Fe(1,2-C_2_B_9_H_10_)_2_-κ^2^-S,S′]} (**8**) was synthesized starting from the 8,8′-bis(methylsulfanyl) derivative of iron bis(dicarbollide) Cs[7] and [(COD)_2_Rh](BF_4_) (Scheme 5).

## 3. Materials and Methods

### 3.1. General Methods

Cobalt bis(dicarbollide) (Me_3_NH)[1] and Cs[1] [19], and iron bis(dicarbollide) Cs[7] [20] derivatives, [(Ph_3_P)_2_PdCl_2_] [36] and [(COD)_2_Rh](BF_4_) [37] were prepared according to the literature procedures. Acetonitrile and dichloromethane were dried using standard procedures [38]. All other chemical reagents were purchased from Sigma Aldrich, Acros Organics and ABCR and used without purification. The reaction progress was monitored by thin layer chromatography (Merck F254 silica gel on aluminum plates) and visualized using 0.5% PdCl_2_ in 1% HCl in aq. MeOH (1:10). Acros Organics silica gel (0.060–0.200 mm) was used for column chromatography. The NMR spectra at 400 MHz (^1^H), 128 MHz (^11^B) and 100 MHz (^13^C) were recorded with Bruker Avance 400 spectrometer. The residual signal of the NMR solvent relative to tetramethylsilane was taken as the internal reference for ^1^H and ^13^C-NMR spectra. ^11^B-NMR spectra were referenced using BF_3_·Et_2_O as external standard. Chemical analyses were performed at the Laboratory of Microanalysis of A. N. Nesmeyanov Institute of Organoelement Compounds.

Single crystal X-ray diffraction experiments for compounds **5** and **6** were carried out using SMART APEX2 CCD diffractometer (λ(Mo-Kα) = 0.71073 Å, graphite monochromator, *ω*-scans) at 120 K. Collected data were processed by the SAINT and SADABS programs incorporated into the APEX2 program package [39]. The structures were solved by the direct methods and refined by the full-matrix least-squares procedure against *F*^2^ in anisotropic approximation. The refinement was carried out with the SHELXTL program [40]. The CCDC numbers (2045460, 2045461, for **5** and **6**, respectively) contain the supplementary crystallographic data for this paper. These data can be obtained free of charge via www.ccdc.cam.ac.uk/data_request/cif.

### 3.2. Synthesis of Complex **2**

Solution of copper(I) iodide (40 mg, 0.21 mmol) in 20 mL of acetonitrile was added to orange solution of ((Me_3_NH)[1]) (100 mg, 0.21 mmol) in 10 mL of acetonitrile and the reaction mixture was stirred at room temperature overnight. Thereafter, the purple reaction mixture was filtered and concentrated under reduced pressure. The crude product was purified by column chromatography on silica using a mixture of dichloromethane and acetone (1:1, *v*/*v*) as eluent to give an purple solid **2** (76 mg, yield 65%). ^1^H-NMR (400 MHz, acetone-*d*_6_): δ 4.43 (2H, br s, C*H_Carb_*), 4.00 (2H, br s, C*H_Carb_*), 2.87 (2H, s, *H_2_*O), 2.21 (6H, m, SC*H_3_*), 2.09 (6H, s, *(CH_3_*)_2_CO) ppm; ^11^B-NMR (128 MHz, acetone-*d*_6_): δ 15.8 (2B, s, *B*-S), −0.7 (2B, d, *J* = 128 Hz), −4.8 (4B, d, *J* = 135 Hz), −5.6 (4B, d, *J* = 113 Hz), −15.7 (2B, d, *J* = 157 Hz), −17.1 (2,B d, *J* = 158 Hz), −23.5 (2B, d) ppm; ^13^C-NMR (100 MHz, acetone-*d*_6_): δ 206.3 (*C*=O), 52.0 (*C_Carb_*H), 51.6 (*C_Carb_*H), 30.6 ((*C*H_3_)_2_CO), 19.4 (S*C*H_3_) ppm.

### 3.3. Synthesis of Complex **3**

Solution of silver(I) nitrate (36 mg, 0.21 mmol) in 20 mL of acetonitrile was added to orange solution of (Me_3_NH)[1] (100 mg, 0.21 mmol) in 10 mL of acetonitrile and the reaction mixture was stirred at room temperature overnight. Thereafter, the reaction mixture was filtered through layer of silica and concentrated under reduced pressure to give an orange solid **3** (90 mg, yield 82%). ^1^H-NMR (400 MHz, acetone-*d*_6_): δ 4.34 (4H, br s, C*H_Carb_*), 2.19 (6H, m, SC*H_3_*) ppm; ^11^B-NMR (128 MHz, acetone-*d*_6_): δ 13.9 (2B, s, *B*-S), −0.3 (2B, d, *J* = 154 Hz), −4.4 (4B, d, *J* = 189 Hz), −6.0 (4B, d, *J* = 138 Hz), −16.9 (4B, d, *J* = 135 Hz), −23.5 (2B, d) ppm; ^13^C-NMR (100 MHz, acetone-*d*_6_): δ 53.4 (*C_Carb_*H), 18.3 (S*C*H_3_) ppm.

### 3.4. Synthesis of Complex **4**

Solution of silver(I) nitrate (36 mg, 0.21 mmol) in 20 mL of acetonitrile was added to orange solution of (Me_3_NH)[1] (100 mg, 0.21 mmol) in 10 mL of acetonitrile and the reaction mixture was stirred at ambient temperature overnight. Thereafter, solution of triphenyl phosphine (55 mg, 0.21 mmol) in 10 mL of acetonitrile was added and the reaction mixture was stirred at room temperature for 1 h. Then, the mixture was filtered through layer of silica and concentrated under reduced pressure to give an orange solid **4** (156 mg, yield 92%).^1^H-NMR (400 MHz, acetone-*d*_6_): δ 7.51 (3H, m, C*H_Ar_*), 7.43 (12H, m, C*H_Ar_*), 4.39 (4H, br s, C*H_Carb_*), 1.97 (6H, q, *J*^3^_B,H_ = 3.8 Hz, SC*H*_3_) ppm; ^11^B-NMR (128 MHz, acetone-*d*_6_): δ 12.8 (2B, s, B-S), 0.1 (2B, d, *J* = 154 Hz), −4.5 (4B, d, *J* = 192 Hz), −6.1 (4B, d, *J* = 156 Hz), −17.3 (4B, d, *J* = 150 Hz), −23.7 (2B, d, *J* = 157 Hz) ppm. Elem. Anal.: Calculated for C_24_H_41_AgB_18_CoPS_2_ (%): C, 36.67; H, 5.26; B, 24.75, Found (%): C, 37.12; H, 5.43; B, 24.58.

### 3.5. Synthesis of Complex **5**

Solution of silver(I) nitrate (36 mg, 0.21 mmol) in 20 mL of acetonitrile was added to orange solution of (Me_3_NH)[1] (100 mg, 0.21 mmol) in 10 mL of acetonitrile and the reaction mixture was stirred at room temperature overnight. Thereafter, solution of [(Ph_3_P)_2_PdCl_2_] (147 mg, 0.21 mmol) in 10 mL of acetonitrile was added and the reaction mixture was stirred at room temperature for 1 h. Then, the reaction mixture was filtered and concentrated under reduced pressure. The crude product was purified by column chromatography on silica using dichloromethane as eluent to give a dark orange solid **5** (70 mg, yield 41%). ^1^H-NMR (400 MHz, acetone-*d*_6_): δ 7.79 (6H, dd, *J*^3^_P,H_ = 11.7 Hz, *J*^3^_H,H_ = 7.6 Hz, C*H_Ar_*), 7.61 (3H, dt, *J*^3^_H,H_ = 7.6 Hz, *J*^5^_P,H_ = 2.0 Hz, C*H_Ar_*), 7.53 (3H, dt, *J*^3^_H,H_ = 7.6 Hz, *J*^4^_P,H_ = 2.2 Hz, C*H_Ar_*), 4.39 (4H, br s, C*H_Carb_*), 2.18 (3H, m, SC*H_3_*), 1.91 (3H, m, SC*H_3_*) ppm; ^11^B-NMR (128 MHz, acetone-*d*_6_): δ 15.1 (1B, s, *B*-S), 11.2 (1B, s, B-S), 2.1 (1B, d), −0.1 (1B, d), −4.5 (8B, d),−15.1 (4B, d), −22.3 (2B, d) ppm. Elem. Anal.: Calculated for C_24_H_41_B_18_ClCoPPdS_2_ (%): C, 35.15; H, 5.04; B, 23.73, Found (%): C, 35.06; H, 5.13; B, 23.88. *Crystallographic data:* C_24_H_41_B_18_ClPCoPdS_2_ are orthorhombic, space group *Pna*2_1_: *a* = 23.3258(8) Å, *b* = 10.3830(3) Å, *c* = 14.8523(5) Å, *V* = 3597.1(2) Å^3^, *Z* = 4, *M* = 820.02, *d*_cryst_ = 1.514 g⋅cm^−3^. *wR*2 = 0.0621 calculated on *F*^2^*_hkl_* for all 10,121 independent reflections with 2*θ* < 59.3°, (GOF = 1.018, *R* = 0.0285 calculated on *F_hkl_* for 9071 reflections with *I* > 2*σ*(*I*)).

### 3.6. Synthesis of Complex **6**

[(COD)_2_Rh](BF_4_) (80 mg, 0.20 mmol) was added to Cs[1] (100 mg, 0.18 mmol) in 4 mL of dichloromethane and the reaction mixture was stirred at room temperature overnight. Thereafter, the red reaction mixture was filtered and concentrated under reduced pressure. The crude product was purified by column chromatography on silica using of chloroform as eluent to give a red solid **6** (90 mg, yield 80%). ^1^H-NMR (400 MHz, acetone-*d*_6_): δ 4.36 (4H, br s, *H*C = C*H*), 4.31 (4H, br s, C*H_Carb_*), 2.45 (4H, m, C*H_2_*), 2.07 (6H, m, SC*H_3_*), 2.05 (m, C*H_2_,* overlap with acetone residual peak) ppm; ^11^B-NMR (128 MHz, acetone-*d*_6_): δ 13.4 (2B, s, *B*-S), 0.5 (2B, d, *J* = 148 Hz), −5.3 (8B, d, *J* = 132 Hz), −15.8 (4B, d, *J* = 157 Hz), −22.7 (2B, d, *J* = 141 Hz) ppm; ^13^C-NMR (100 MHz, acetone-*d*_6_): δ 87.0 (d, *J*^1^_C,Rh_ = 11.6 Hz, H*C* = *C*H), 53.0 (*C_Carb_*H), 31.4 (*C*H_2_), 19.0 (S*C*H_3_) ppm. Elem. Anal.: Calculated for C_14_H_38_B_18_CoRhS_2_ (%): C, 26.82; H, 6.11; B, 31.03, Found (%): C, 26.14; H, 5.78; B, 30.32. *Crystallographic data*: C_14_H_38_B_18_CoRhS_2_·CHCl_3_ are monoclinic, space group *P2_1_*/*c*: *a* = 14.5739(14) Å, *b* = 33.265(3) Å, *c* = 13.1138(12) Å, *β* = 99.296(2)°, *V* = 6274.0(10) Å^3^, Z = 8, M = 746.35, *d*_cryst_ = 1.580 g·cm^−3^. *wR*2 = 0.1327 calculated on *F*^2^*_hkl_* for all 13,854 independent reflections with 2*θ* < 54.3*θ*, (GOF = 1.009, *R* = 0.0568 calculated on *F_hkl_* for 8164 reflections with *I* > 2*σ*(*I*)).

### 3.7. Synthesis of Complex **8**

[(COD)_2_Rh](BF_4_) (200 mg, 0.49 mmol) was added to Cs[7] (270 mg, 0.49 mmol) in 10 mL of dichloromethane and the reaction mixture was stirred at room temperature overnight. Thereafter, the brown mixture was filtered and concentrated under reduced pressure. The crude product was purified twice by column chromatography on silica with use of chloroform (first column) and toluene (second column) as eluent to give a brown solid **8** (190 mg, yield 62%). ^1^H-NMR (400 MHz, acetone-*d*_6_): δ 130.13 (br, B*H*/C*H_Carb_*), 46.56 (br, B*H*), 3.78 (br, B*H*), 1.30 (br, B*H*), 0.11 (br s, SC*H_3_*), −0.54 (br s, C*H_2_*), −1.01 (br s, C*H_2_*), −4.95 (br s, *H*C = C*H*), −10.66 (br, B*H*) ppm; ^11^B-NMR (128 MHz, acetone-*d*_6_): δ 108.6, 14.8, 5.5, −21.9, −345.1 (*B8*-S + *B4* + *B7*) ppm; ^13^C-NMR (100 MHz, acetone-*d*_6_): δ 80.6 (d, *J*^1^_C,Rh_ = 8.9 Hz, H*C* = *C*H), 25.9 (*C*H_2_), −21.0 (m, S*C*H_3_) ppm. Elem. Anal.: Calculated for C_14_H_38_B_18_CoRhS_2_ (%): C, 26.95; H, 6.14; B, 31.19, Found (%): C, 26.37; H, 6.02; B, 30.98.

## 4. Conclusions

The complexation of the 8,8′-bis(methylsulfanyl) derivatives of cobalt and iron bis(dicarbollides) [8,8′-(MeS)_2_-3,3′-M(1,2-C_2_B_9_H_10_)_2_]^−^ (M = Co, Fe) with various transition metals (copper, silver, palladium, rhodium) leads to the formation of the corresponding chelate complexes, which is accompanied by a transition from the *transoid* to the *cisoid* conformation of the bis(dicarbollide) complex. This transition is reversible on removal of the external transition metal using stronger ligands or precipitating agents and can be used in design of coordination-driven molecular switches based on transition metal bis(dicarbollide) complexes.

## Supplementary Materials

The following are available online: NMR spectra of compounds **2**–**6** and **8**.
