# Supplementary material for: Bis(dicarbollide) Complexes of Transition Metals as a Platform for Molecular Switches. Study of Complexation of 8,8′-Bis(methylsulfanyl) Derivatives of Cobalt and Iron Bis(dicarbollides)
Source: Molecules. 2020 Dec 5;25(23):5745. doi: 10.3390/molecules25235745 (PMC7730339; doi:10.3390/molecules25235745)
Supplement: Supplementary file 1 [file molecules-25-05745-s001.zip › molecules-1028383-supplementary/Molecules-SI.pdf]

SUPPORTING INFORMATION

Bis(Dicarbollide) Complexes of Transition Metals as a Platform for Molecular Switches. Study of Complexation of 8,8′-Bis(Methylsulfanyl) Derivatives of Cobalt and Iron Bis(Dicarbollides)

Sergey A. Anufriev, Sergey V. Timofeev, Alexei A. Anisimov, Kyrill Yu. Suponitsky and Igor B. Sivaev

NMR spectra 2

Cu(I) complex with 8,8′-$(SMe)_2$ **Cobalt(III)** *bis*(Dicarbollide) (**2**) 2

Ag(I) complex with 8,8′-$(SMe)_2$ **Cobalt(III)** *bis*(Dicarbollide) (**3**) 6

Ag(I) complex with 8,8′-$(SMe)_2$ **Cobalt(III)** *bis*(Dicarbollide) and **PPh_3** (**4**) 10

Pd(II) complex with 8,8′-$(SMe)_2$ **Cobalt(III)** *bis*(Dicarbollide), **PPh_3** and **Cl^-** (**5**) 13

Rh(I) complex with 8,8′-$(SMe)_2$ **Cobalt(III)** *bis*(Dicarbollide) and **COD** (**6**) 16

Rh(I) complex with 8,8′-$(SMe)_2$ **Iron(III)** *bis*(Dicarbollide) and **COD** (**8**) 22

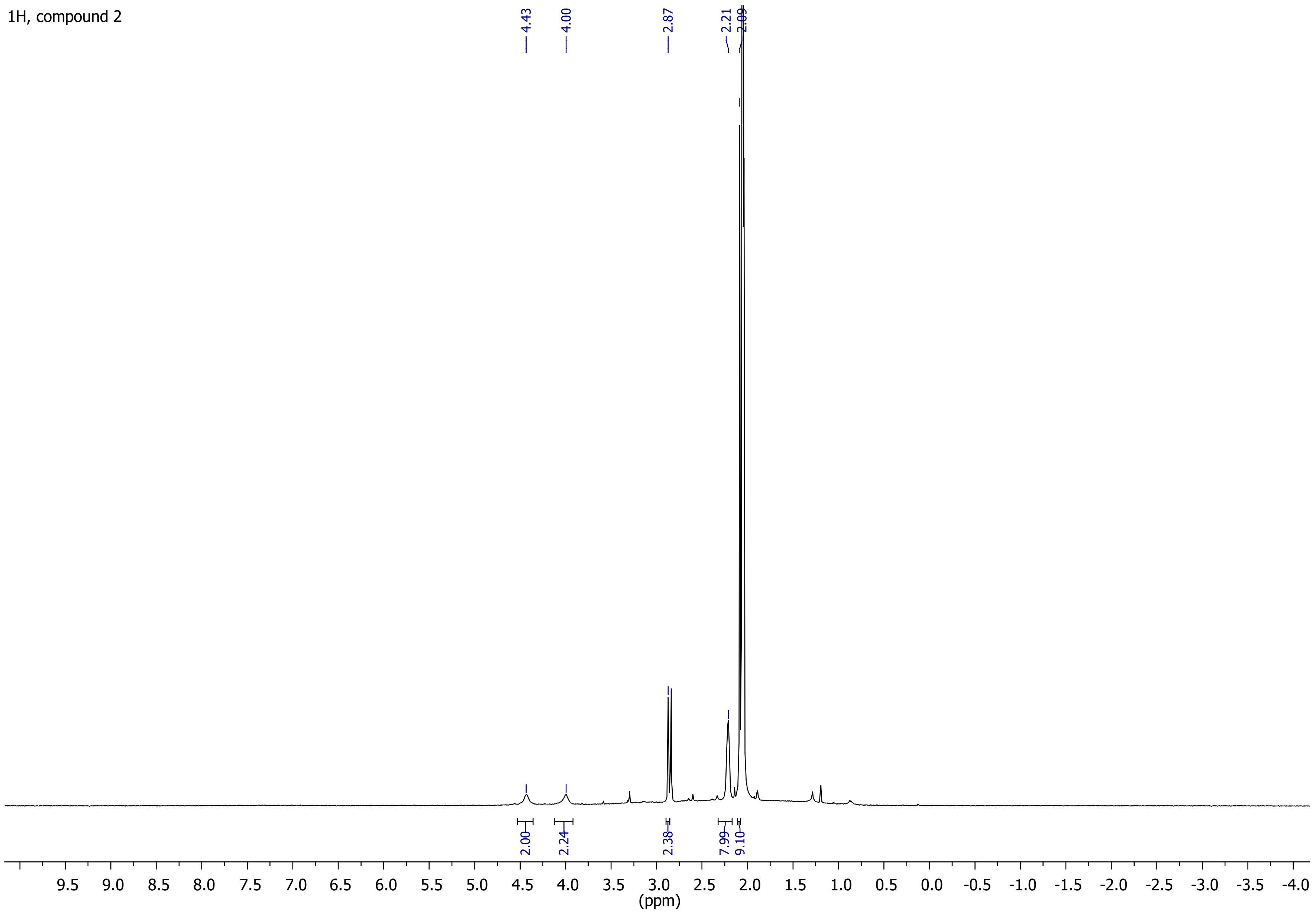
1H, compound 2
4.43
4.00
2.87
2.21
2.09
2.00
2.24
2.38
7.99
9.10
9.5 9.0 8.5 8.0 7.5 7.0 6.5 6.0 5.5 5.0 4.5 4.0 3.5 3.0 2.5 2.0 1.5 1.0 0.5 0.0 -0.5 -1.0 -1.5 -2.0 -2.5 -3.0 -3.5 -4.0
(ppm)

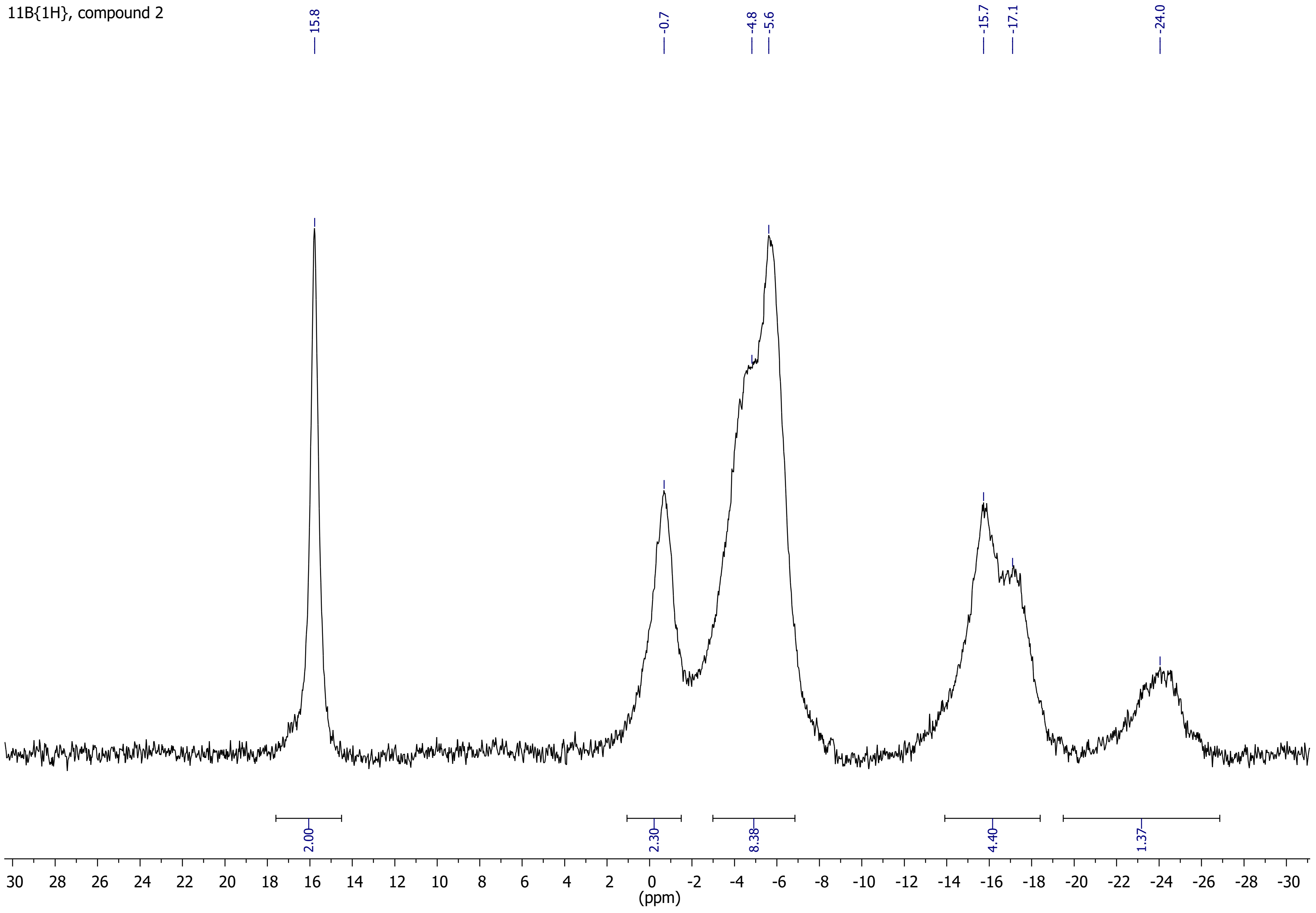
11B{1H}, compound 2
15.8
-0.7
-4.8
-5.6
-15.7
-17.1
-24.0
2.00
2.30
8.38
4.40
1.37
30 28 26 24 22 20 18 16 14 12 10 8 6 4 2 0 -2 -4 -6 -8 -10 -12 -14 -16 -18 -20 -22 -24 -26 -28 -30
(ppm)

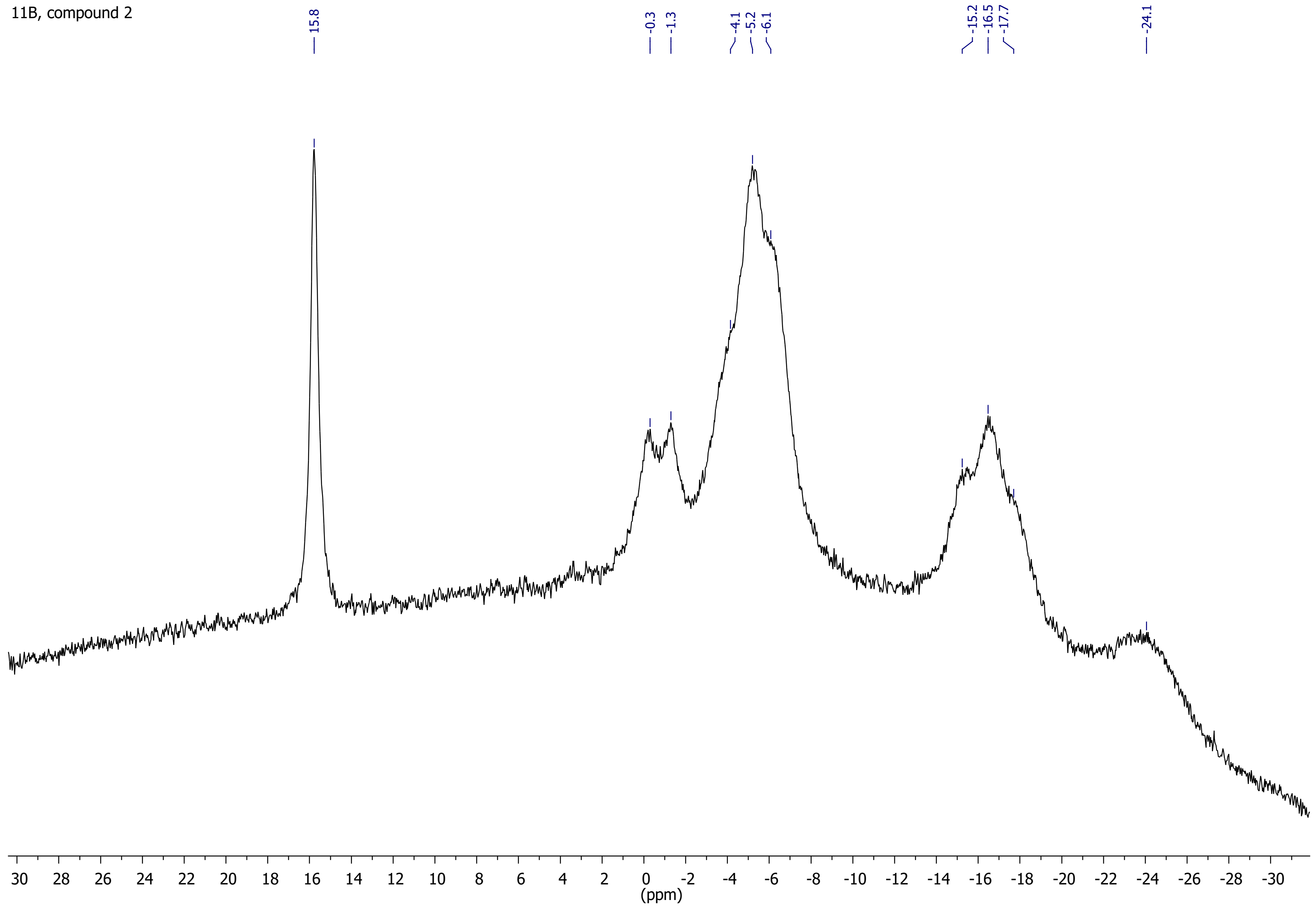
11B, compound 2
15.8
-0.3
-1.3
-4.1
-5.2
-6.1
-15.2
-16.5
-17.7
-24.1
30 28 26 24 22 20 18 16 14 12 10 8 6 4 2 0 -2 -4 -6 -8 -10 -12 -14 -16 -18 -20 -22 -24 -26 -28 -30
(ppm)

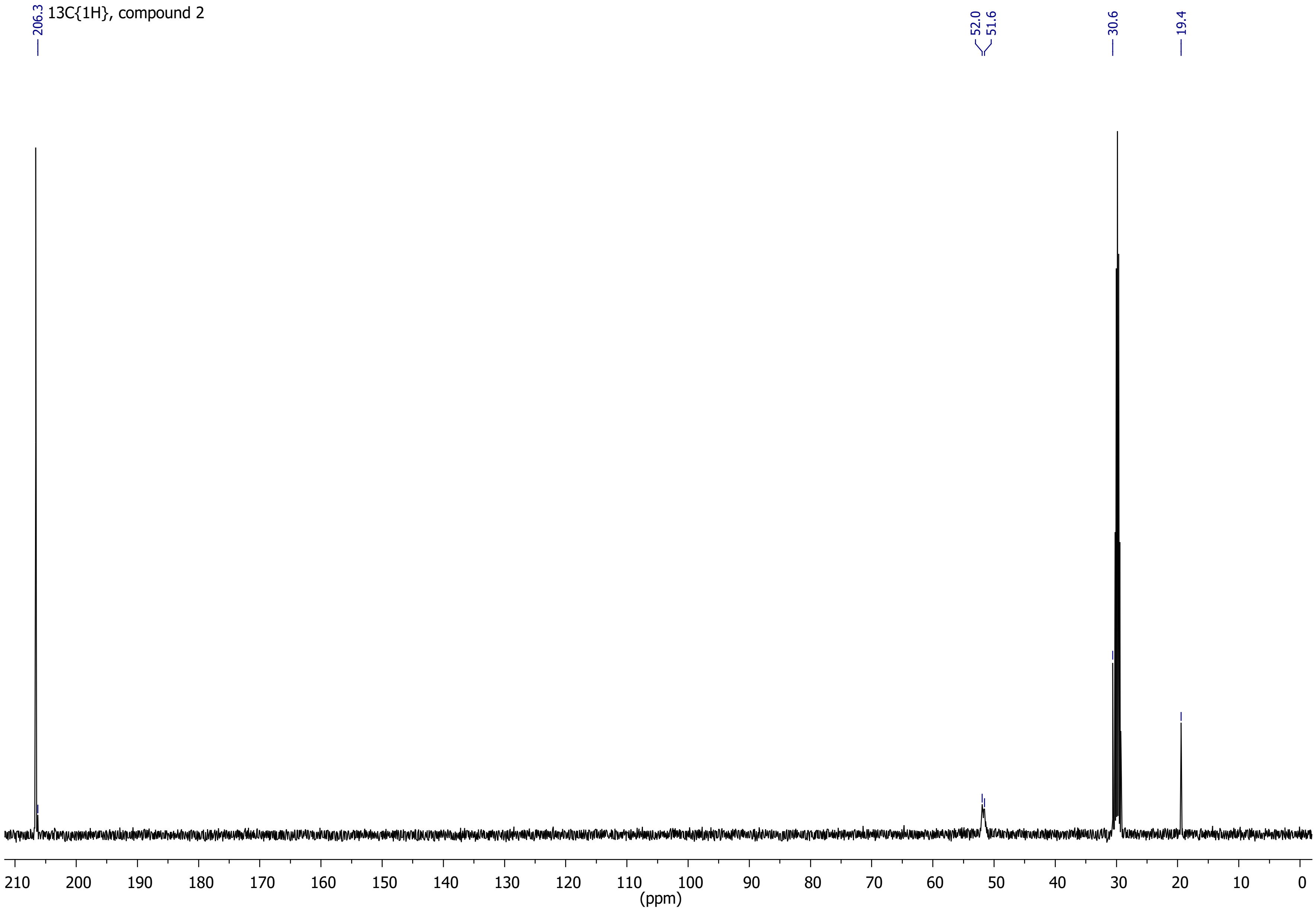

13C{1H}, compound 2
206.3
52.0
51.6
30.6
19.4
210 200 190 180 170 160 150 140 130 120 110 100 90 80 70 60 50 40 30 20 10 0
(ppm)

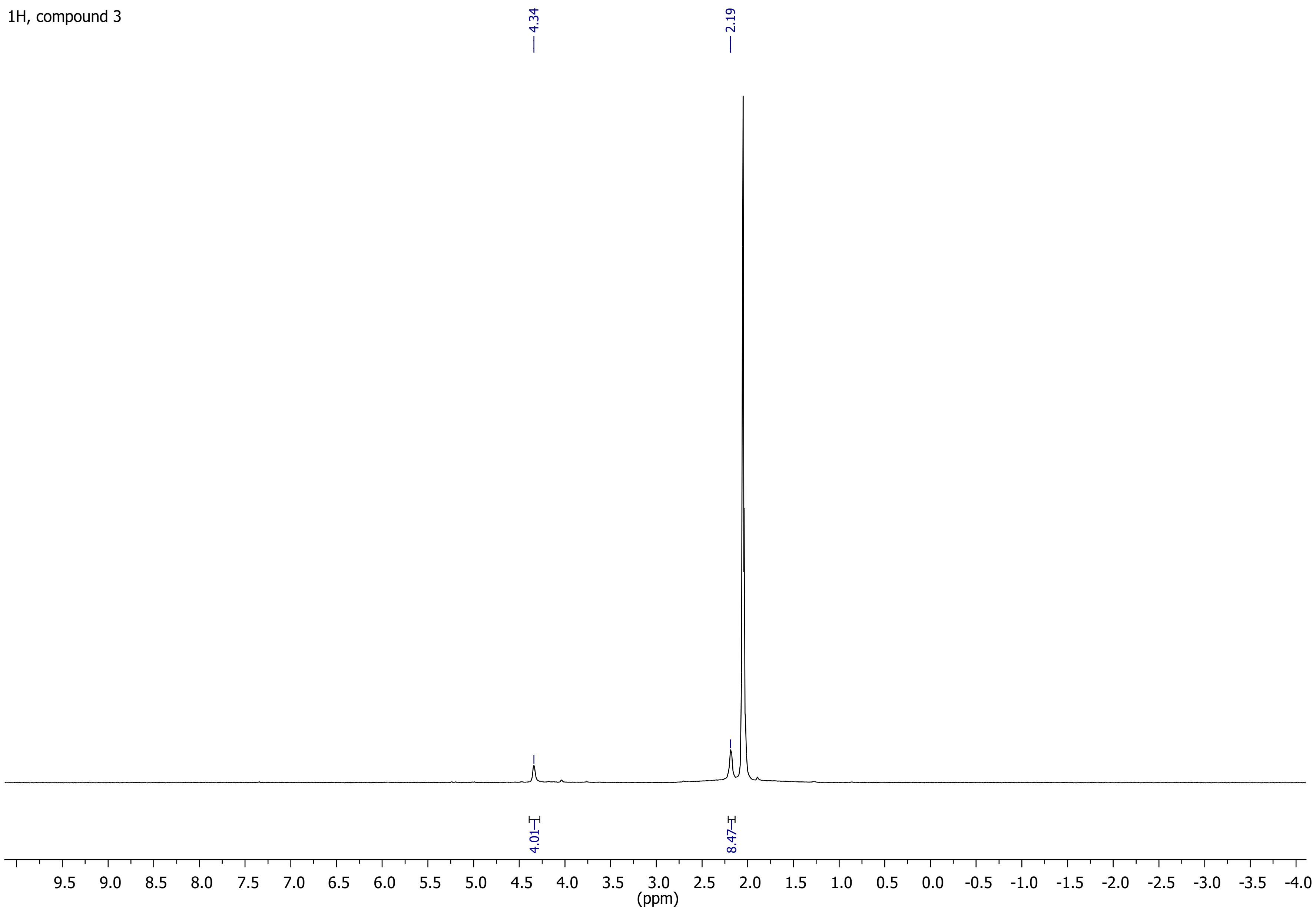
1H, compound 3
4.34
2.19
4.01
8.47
9.5 9.0 8.5 8.0 7.5 7.0 6.5 6.0 5.5 5.0 4.5 4.0 3.5 3.0 2.5 2.0 1.5 1.0 0.5 0.0 -0.5 -1.0 -1.5 -2.0 -2.5 -3.0 -3.5 -4.0
(ppm)

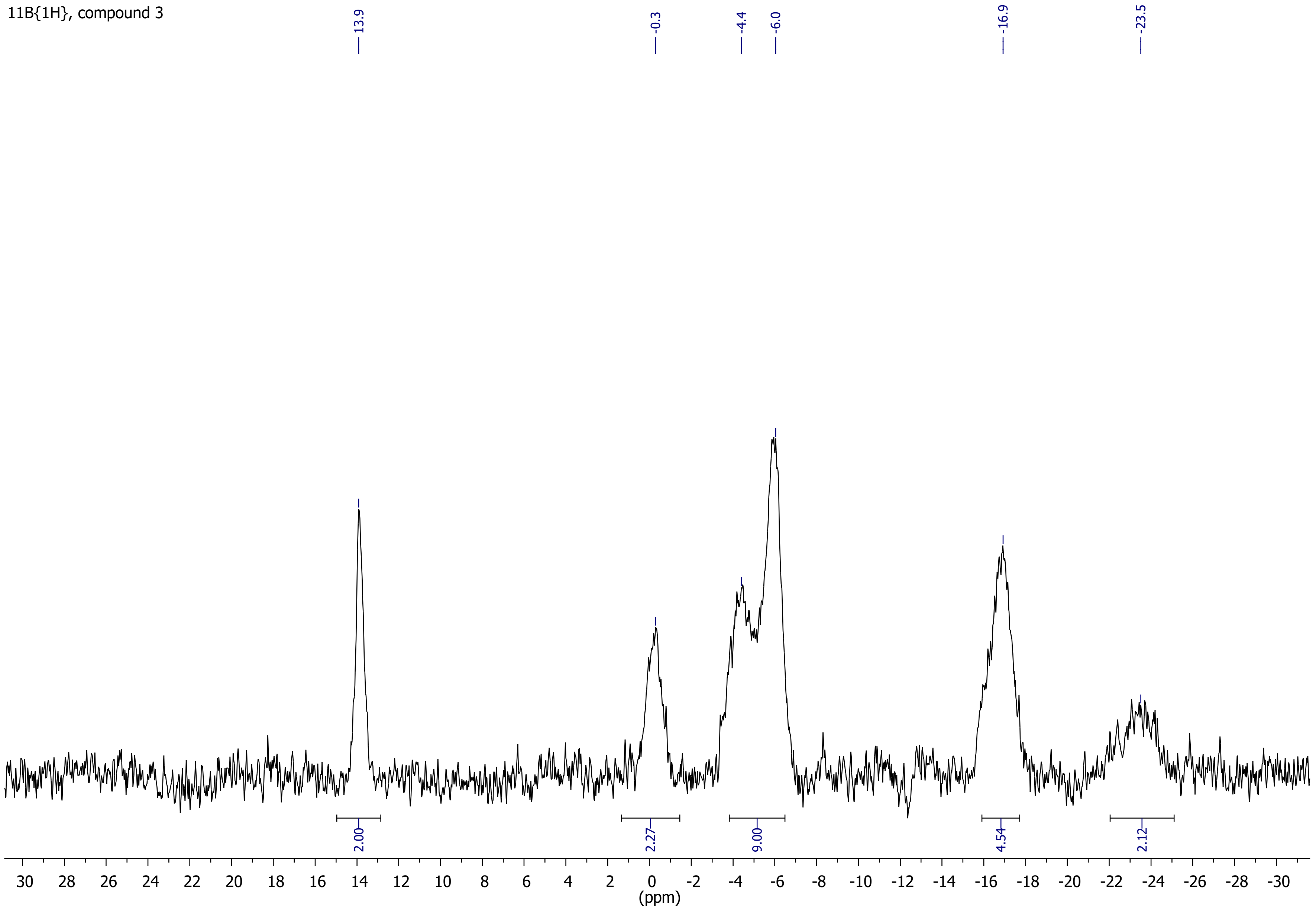

11B{1H}, compound 3
13.9
-0.3
-4.4
-6.0
-16.9
-23.5
2.00
2.27
9.00
4.54
2.12
30 28 26 24 22 20 18 16 14 12 10 8 6 4 2 0 -2 -4 -6 -8 -10 -12 -14 -16 -18 -20 -22 -24 -26 -28 -30
(ppm)

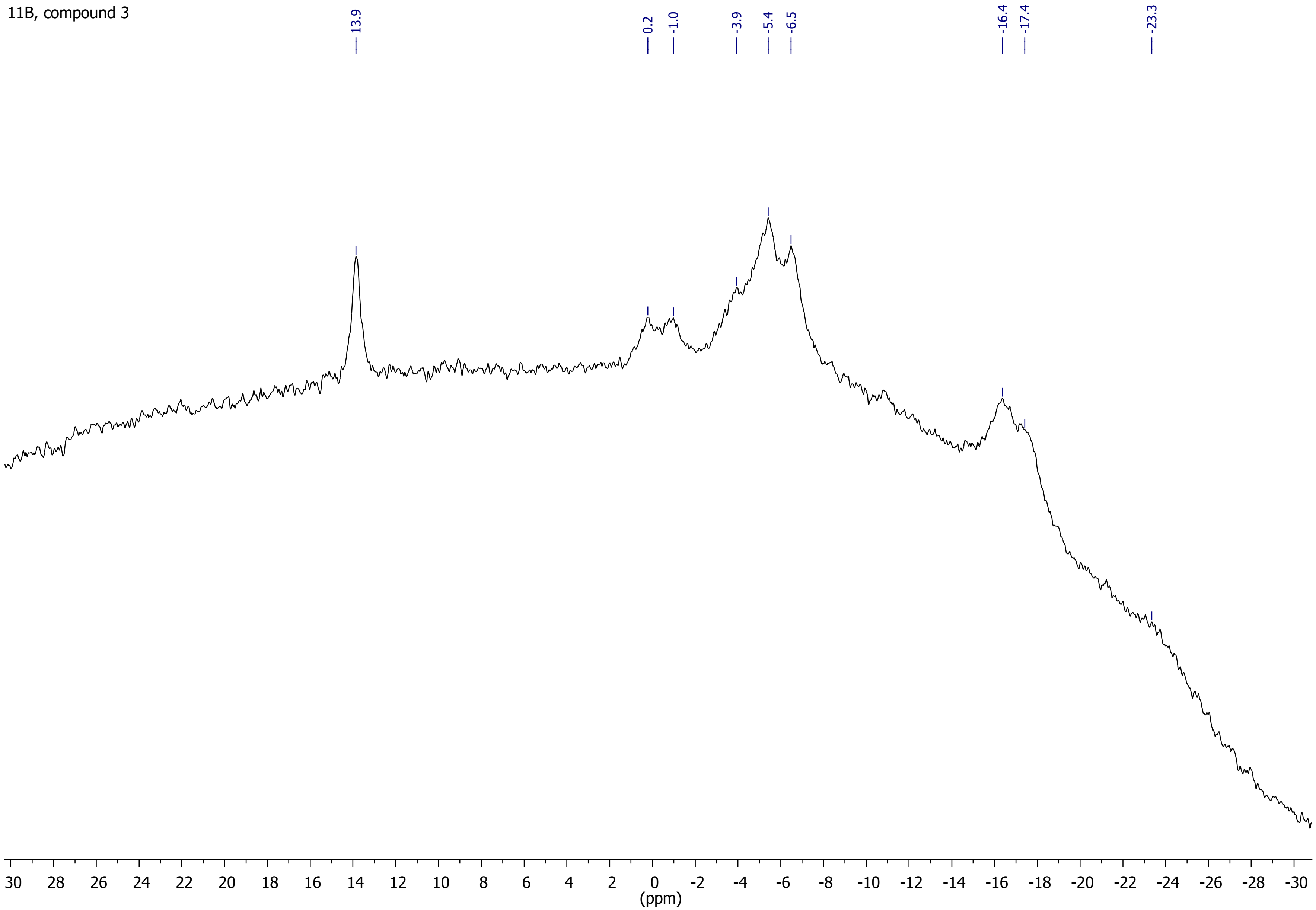
11B, compound 3
13.9
0.2
-1.0
-3.9
-5.4
-6.5
-16.4
-17.4
-23.3
30 28 26 24 22 20 18 16 14 12 10 8 6 4 2 0 -2 -4 -6 -8 -10 -12 -14 -16 -18 -20 -22 -24 -26 -28 -30
(ppm)

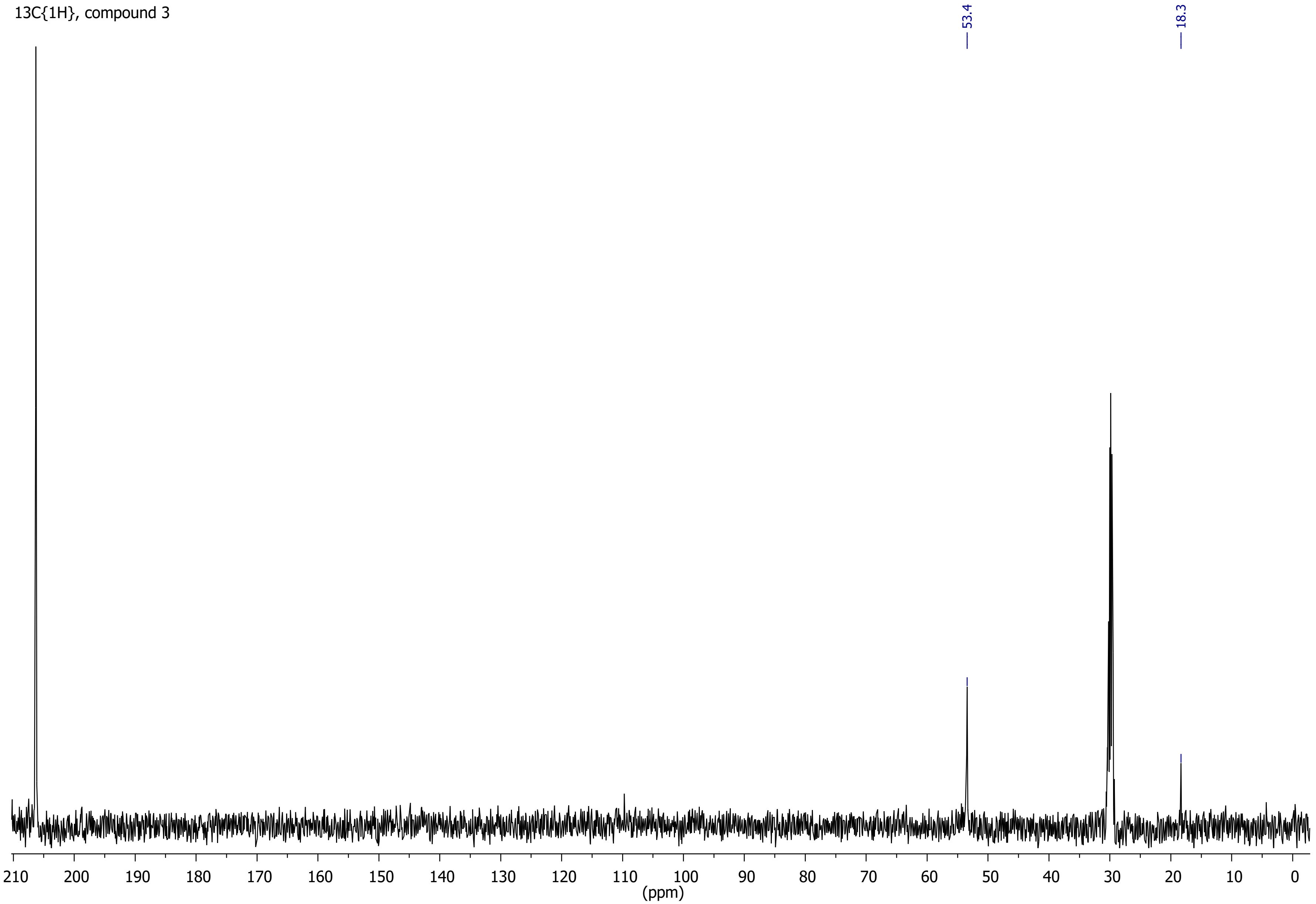

13C{1H}, compound 3
53.4
18.3
210
200
190
180
170
160
150
140
130
120
110
100
90
80
70
60
50
40
30
20
10
0
(ppm)

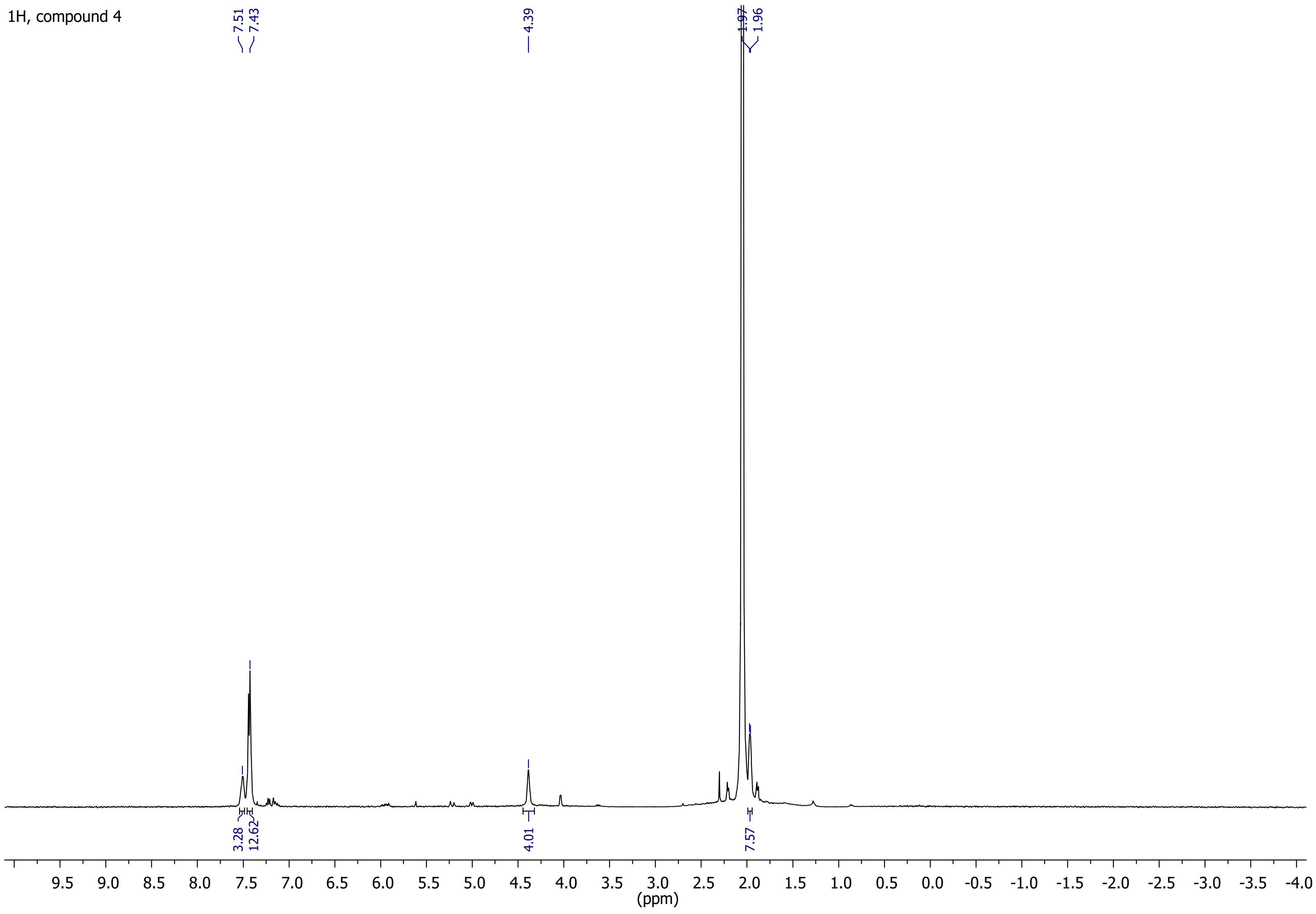

1H, compound 4
7.51
7.43
4.39
1.97
1.96
3.28
12.62
4.01
7.57
9.5 9.0 8.5 8.0 7.5 7.0 6.5 6.0 5.5 5.0 4.5 4.0 3.5 3.0 2.5 2.0 1.5 1.0 0.5 0.0 -0.5 -1.0 -1.5 -2.0 -2.5 -3.0 -3.5 -4.0
(ppm)

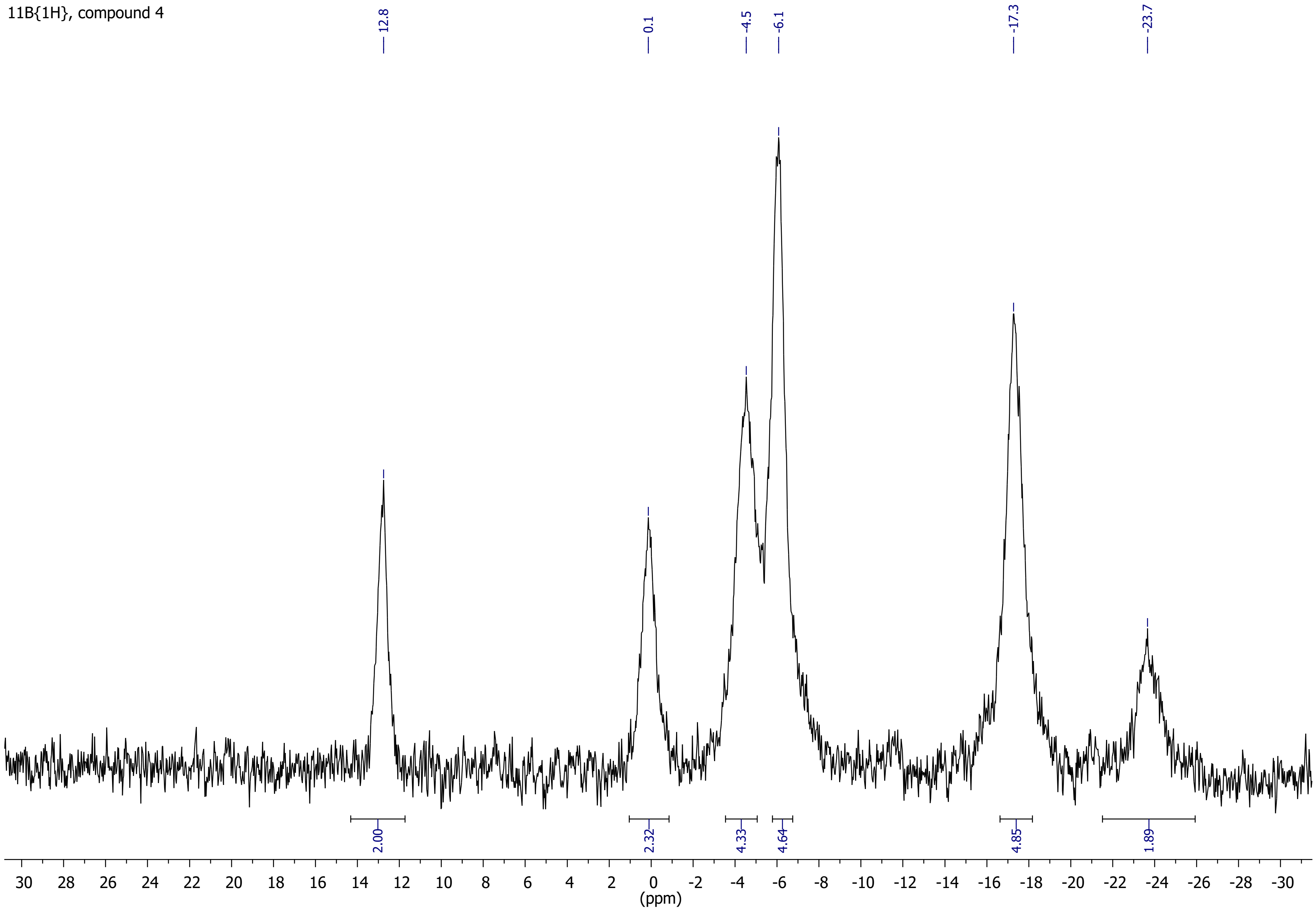
11B{1H}, compound 4
12.8
0.1
-4.5
-6.1
-17.3
-23.7
2.00
2.32
4.33
4.64
4.85
1.89
30 28 26 24 22 20 18 16 14 12 10 8 6 4 2 0 -2 -4 -6 -8 -10 -12 -14 -16 -18 -20 -22 -24 -26 -28 -30
(ppm)

11B, compound 4

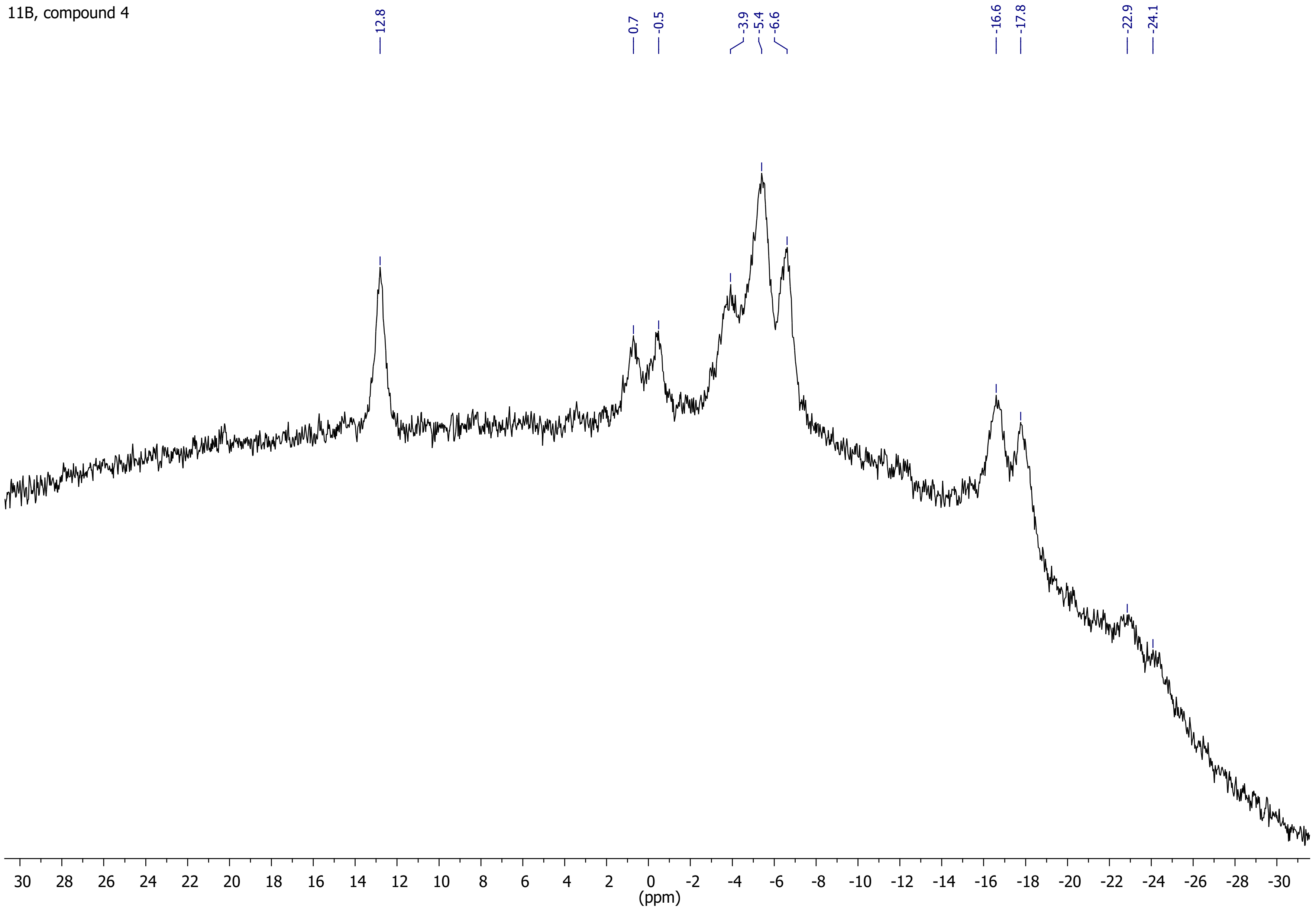

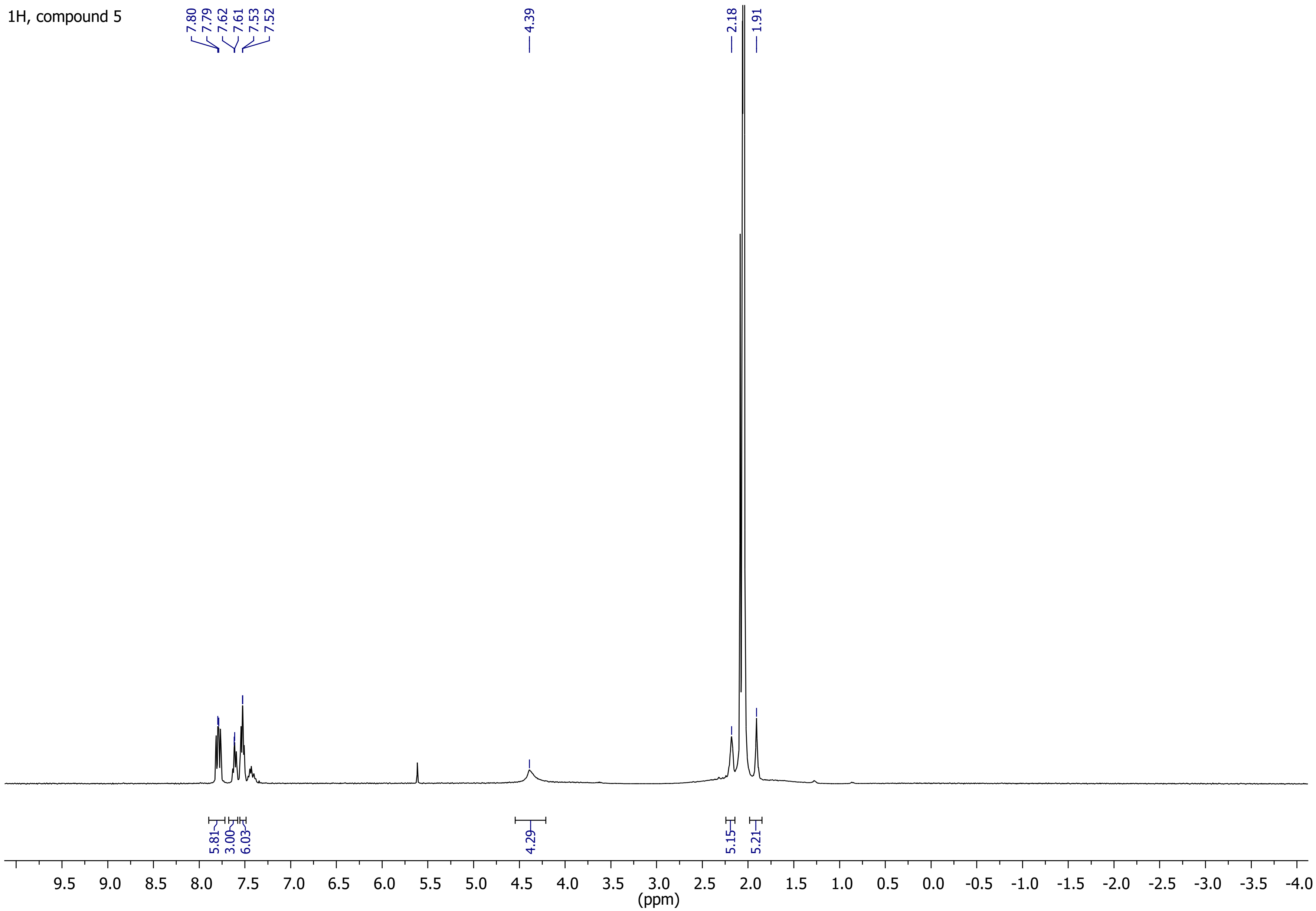

1H, compound 5
7.80
7.79
7.62
7.61
7.53
7.52
4.39
2.18
1.91
5.81
3.00
6.03
4.29
5.15
5.21
9.5 9.0 8.5 8.0 7.5 7.0 6.5 6.0 5.5 5.0 4.5 4.0 3.5 3.0 2.5 2.0 1.5 1.0 0.5 0.0 -0.5 -1.0 -1.5 -2.0 -2.5 -3.0 -3.5 -4.0
(ppm)

11B{1H}, compound 5

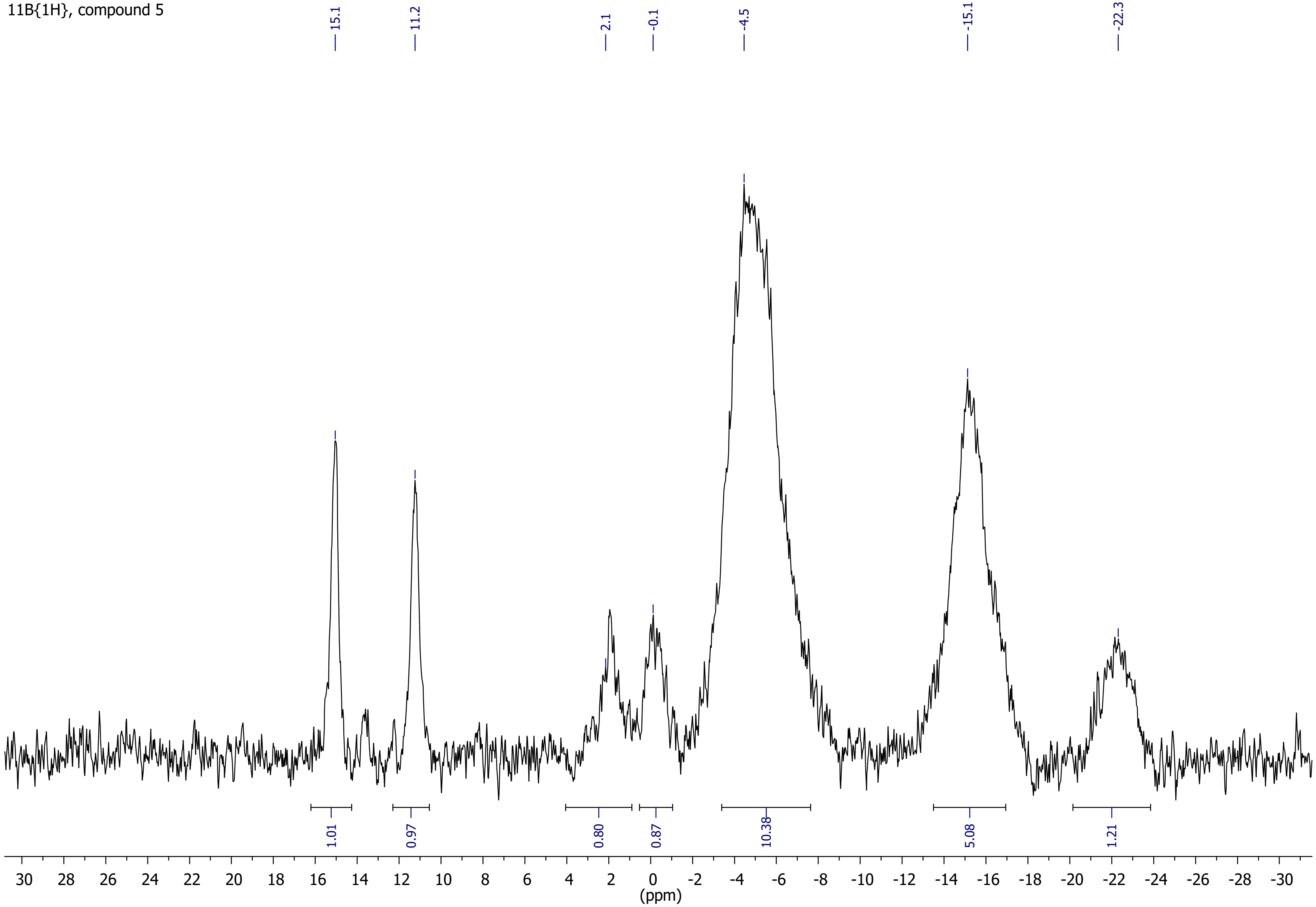

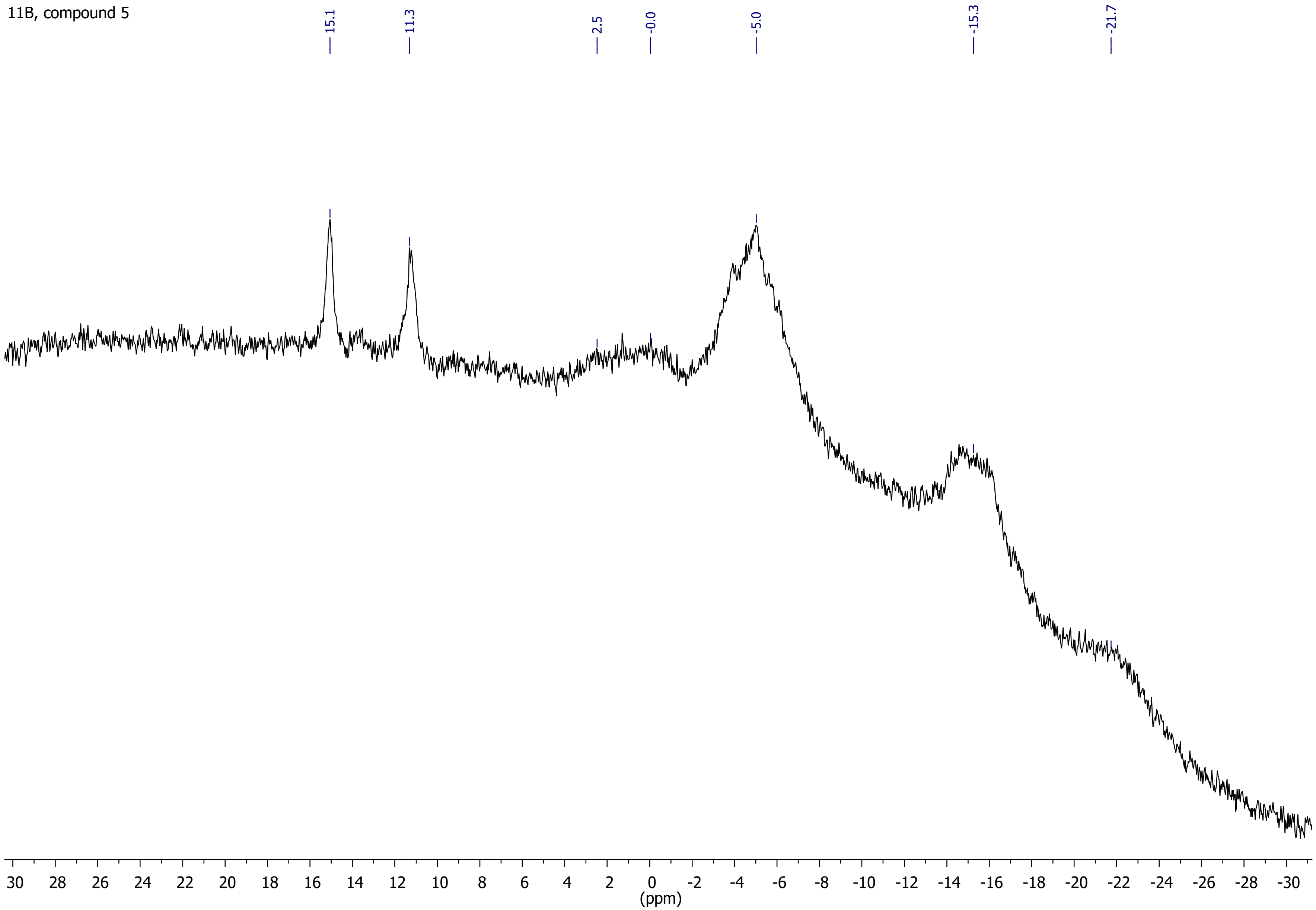
11B, compound 5
15.1
11.3
2.5
-0.0
-5.0
-15.3
-21.7
30 28 26 24 22 20 18 16 14 12 10 8 6 4 2 0 -2 -4 -6 -8 -10 -12 -14 -16 -18 -20 -22 -24 -26 -28 -30
(ppm)

1H, compound 6

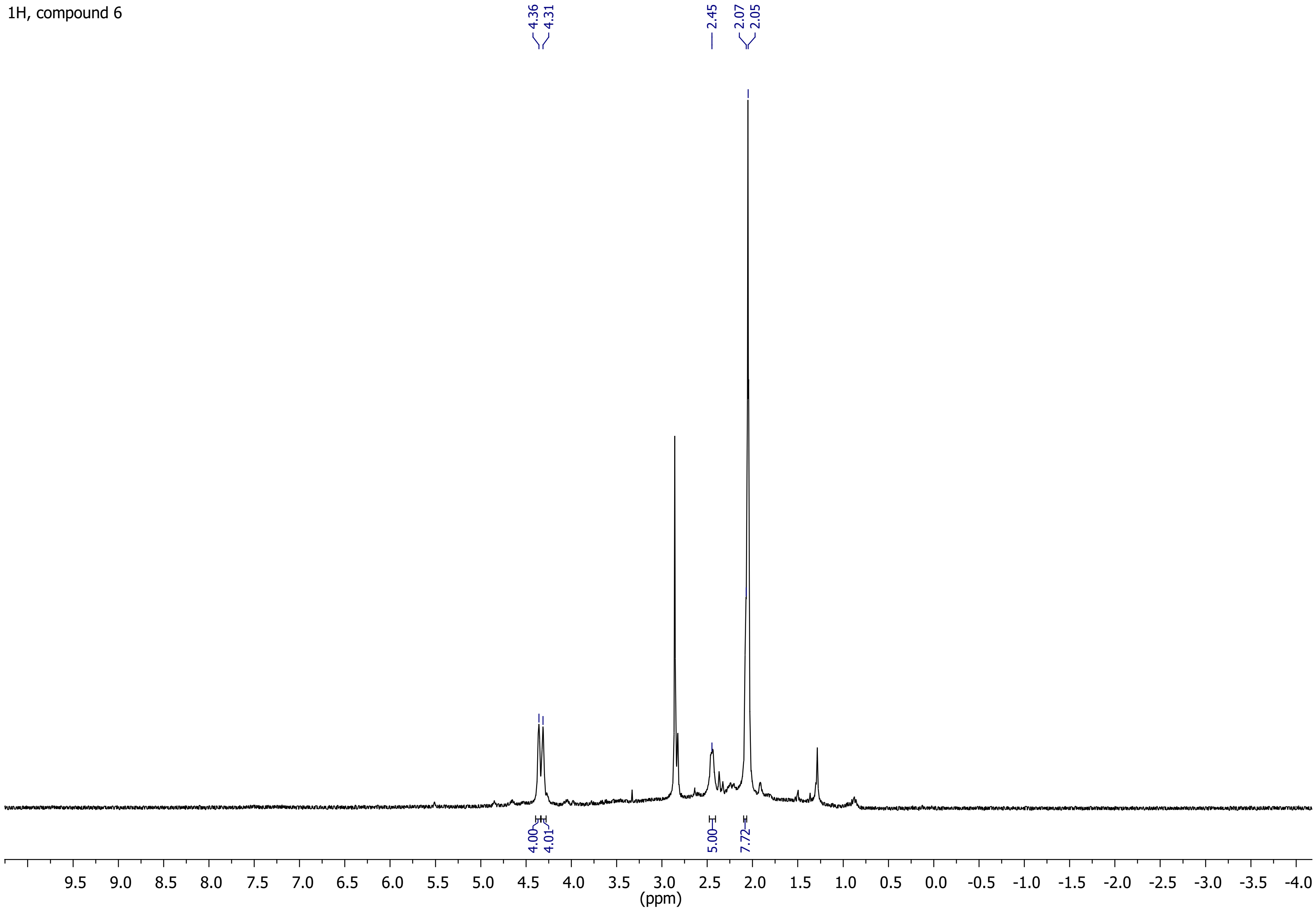

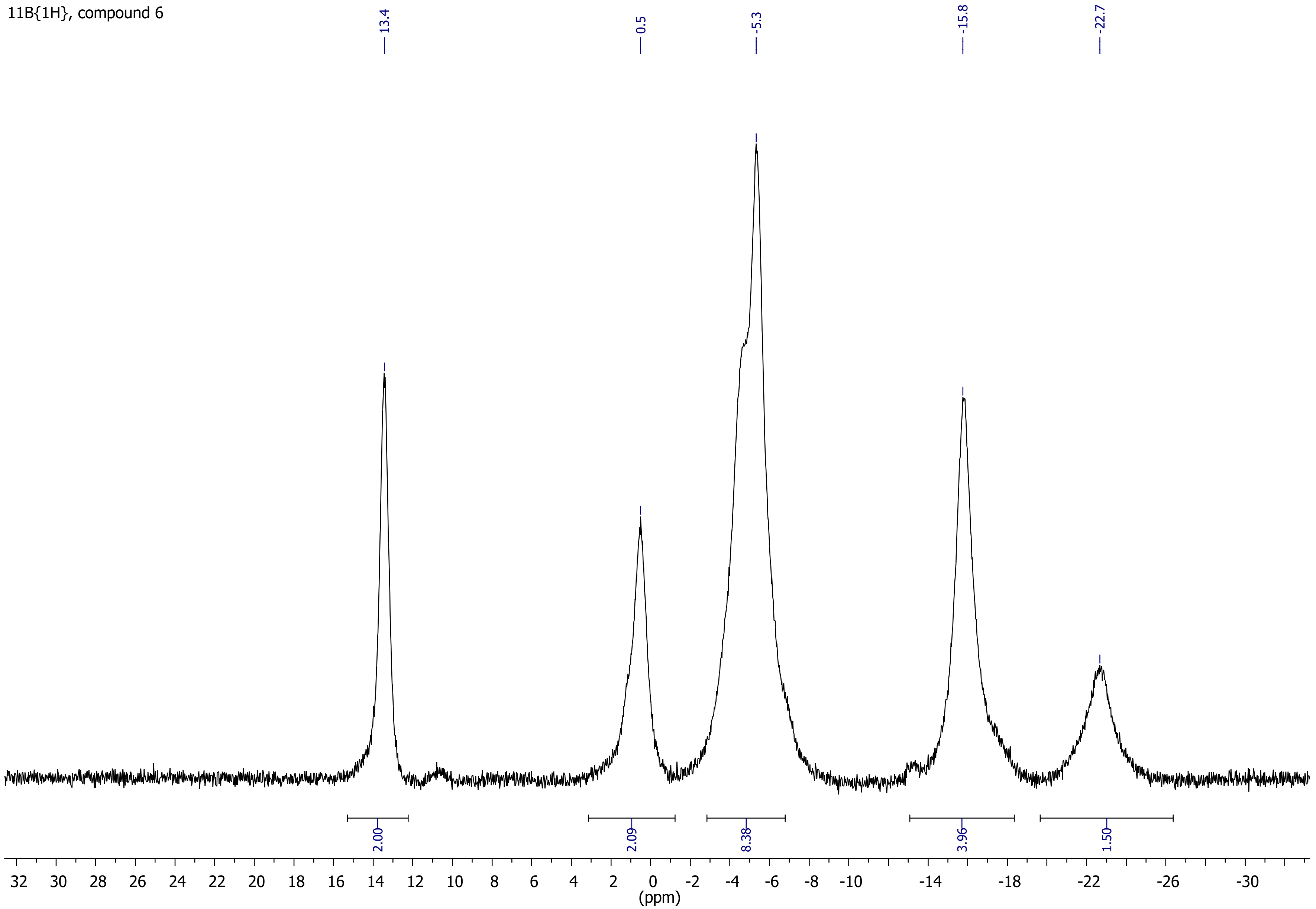
11B{1H}, compound 6
13.4
0.5
-5.3
-15.8
-22.7
2.00
2.09
8.38
3.96
1.50
32 30 28 26 24 22 20 18 16 14 12 10 8 6 4 2 0 -2 -4 -6 -8 -10 -14 -18 -22 -26 -30
(ppm)

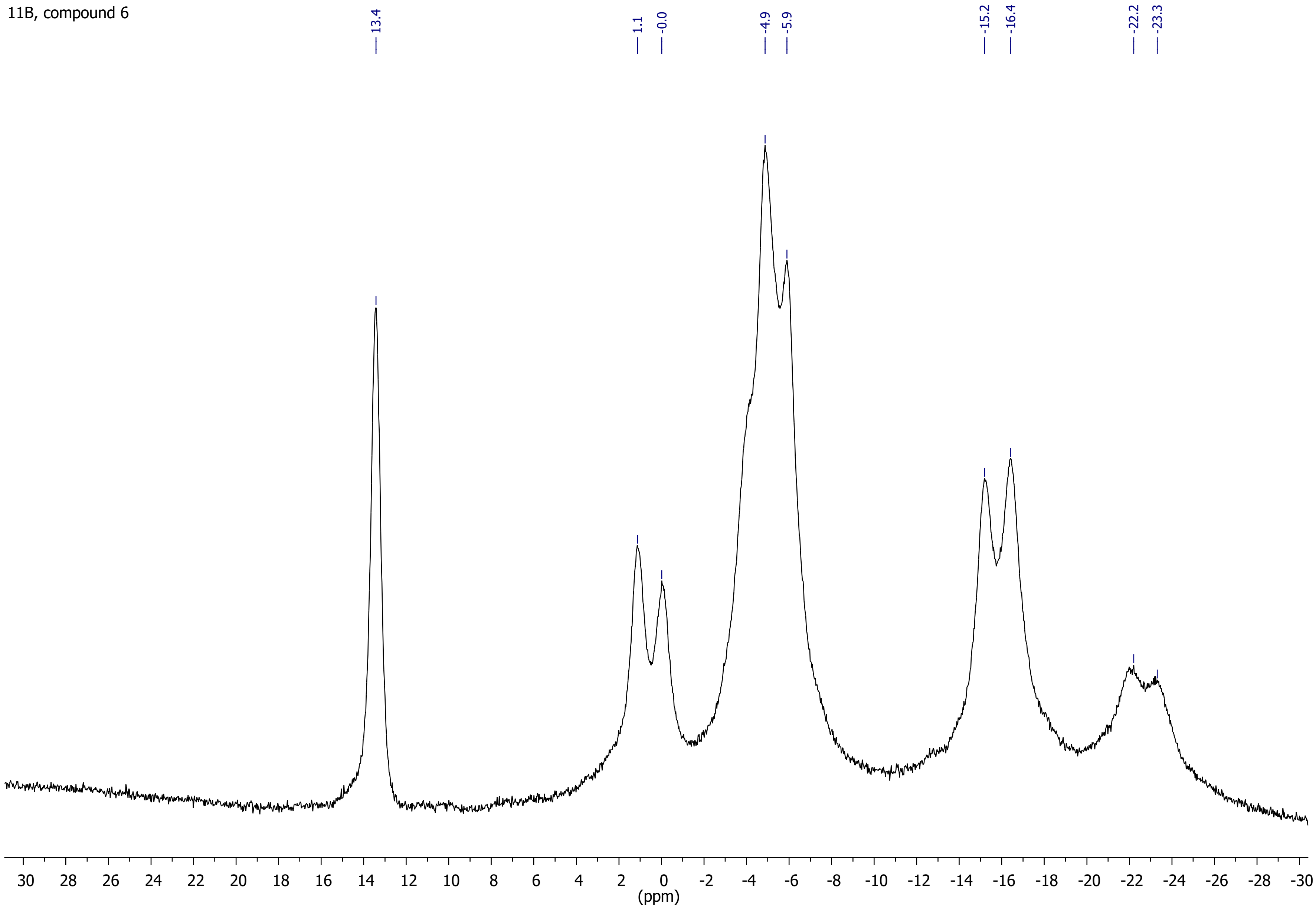
11B, compound 6
13.4
1.1
-0.0
-4.9
-5.9
-15.2
-16.4
-22.2
-23.3
30 28 26 24 22 20 18 16 14 12 10 8 6 4 2 0 -2 -4 -6 -8 -10 -12 -14 -16 -18 -20 -22 -24 -26 -28 -30
(ppm)

13C{1H}, compound 6

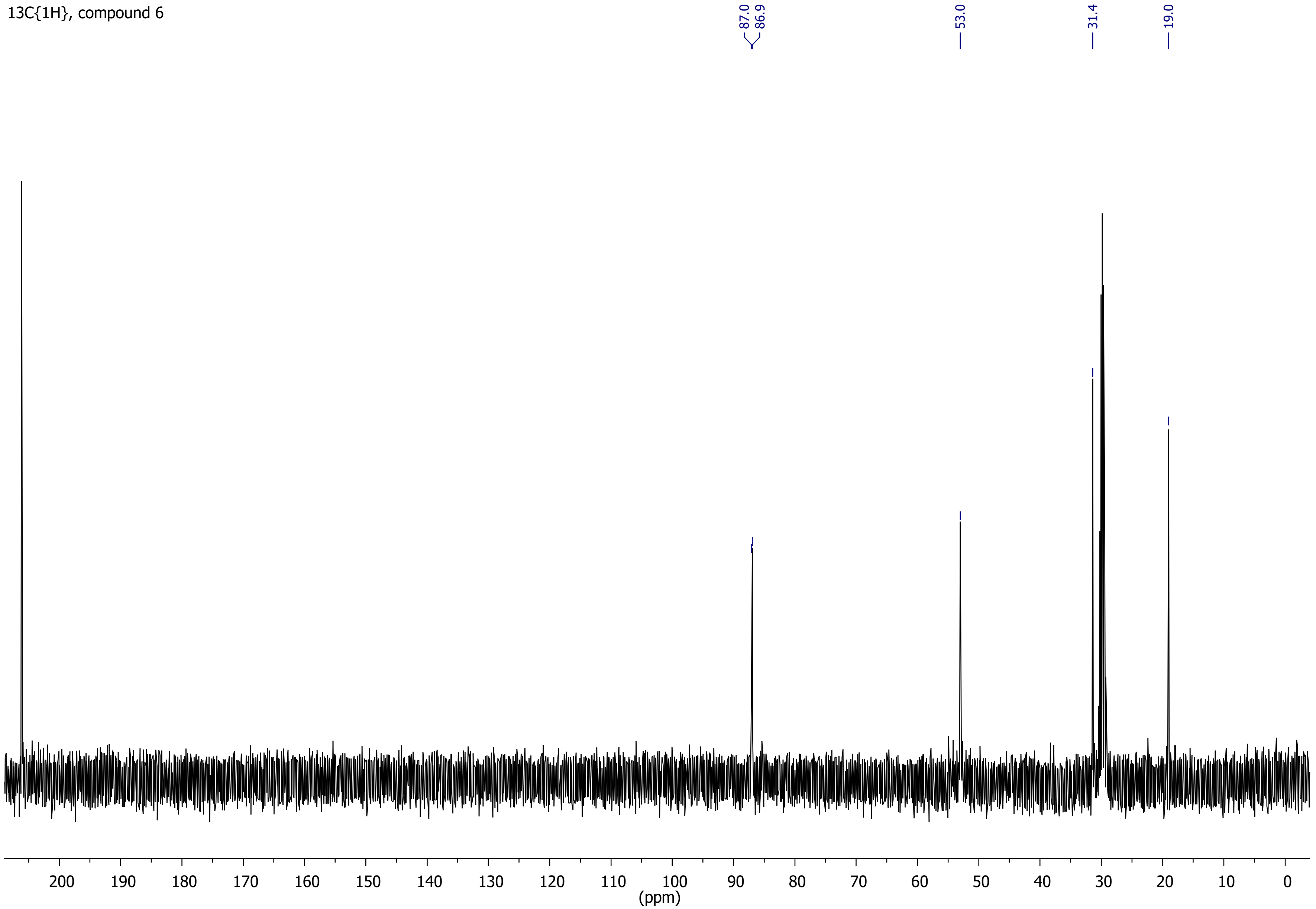

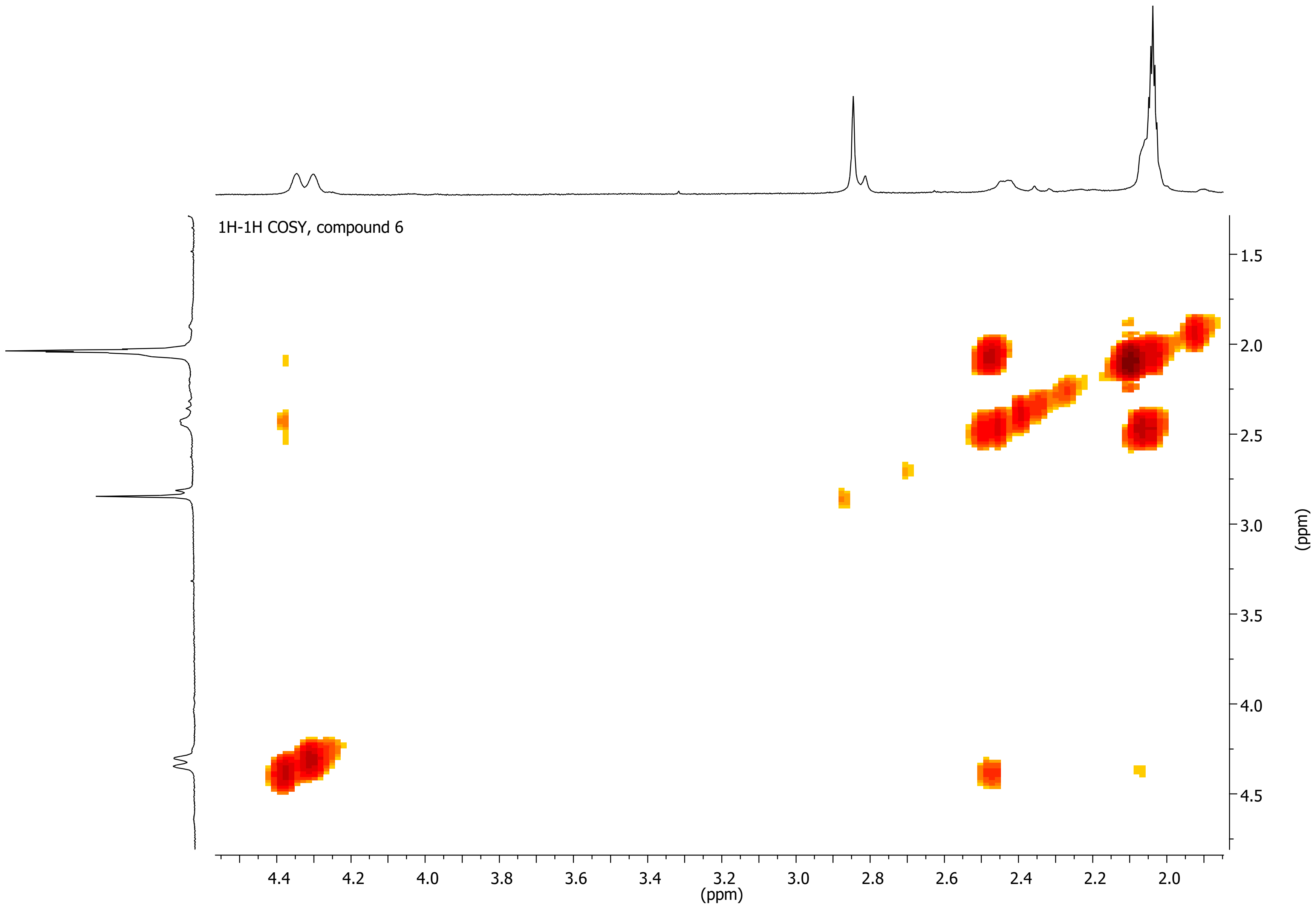
1H-1H COSY, compound 6
4.4
4.2
4.0
3.8
3.6
3.4
3.2
3.0
2.8
2.6
2.4
2.2
2.0
(ppm)
1.5
2.0
2.5
3.0
3.5
4.0
4.5
(ppm)

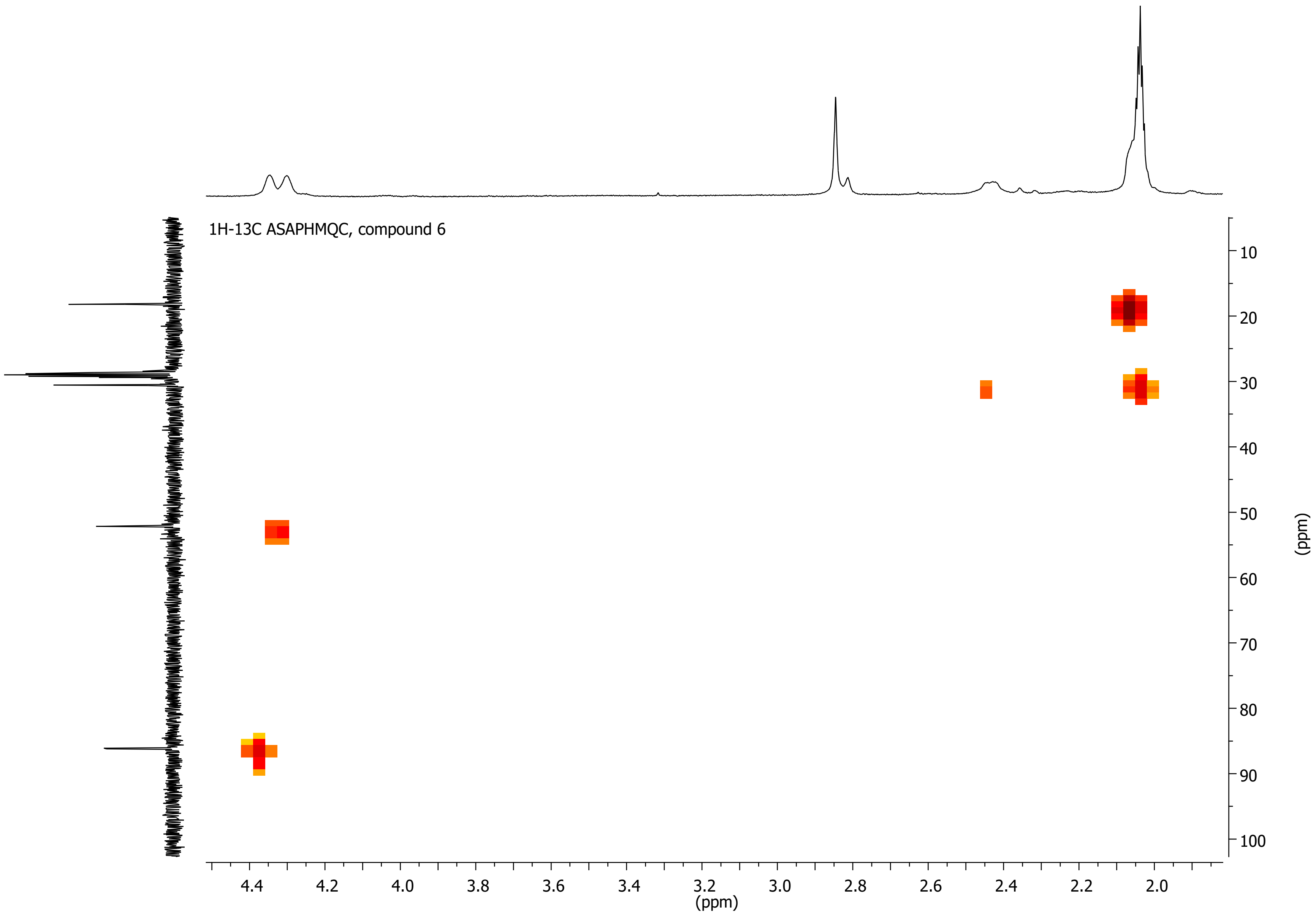
1H-13C ASAPHMQC, compound 6
4.4
4.2
4.0
3.8
3.6
3.4
3.2
3.0
2.8
2.6
2.4
2.2
2.0
(ppm)
10
20
30
40
50
60
70
80
90
100
(ppm)

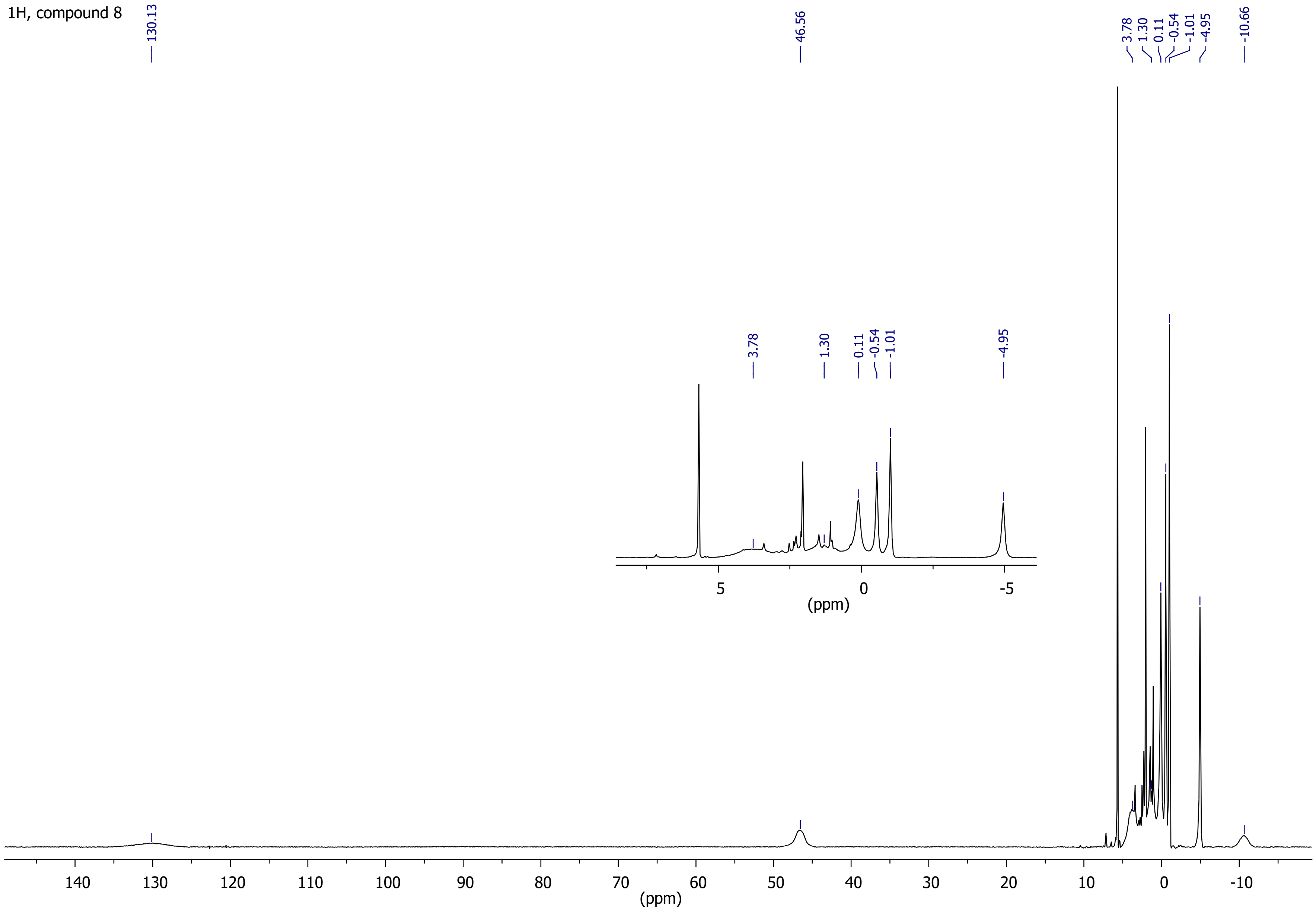

1H, compound 8
130.13
46.56
3.78
1.30
0.11
-0.54
-1.01
-4.95
-10.66
3.78
1.30
0.11
-0.54
-1.01
-4.95
5
0
-5
(ppm)
140
130
120
110
100
90
80
70
60
50
40
30
20
10
0
-10
(ppm)

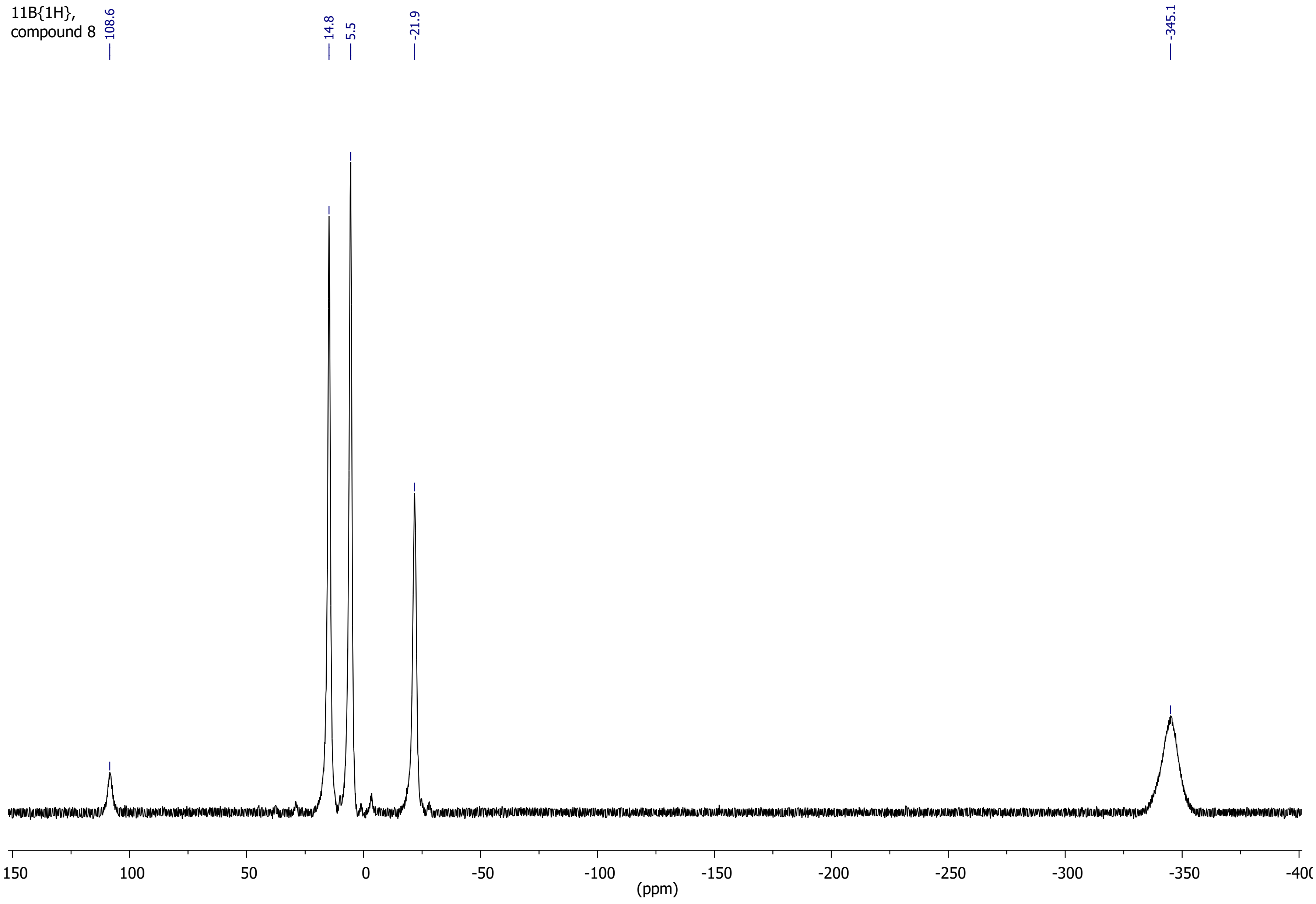
11B{1H},
compound 8
108.6
14.8
5.5
-21.9
-345.1
150
100
50
0
-50
-100
-150
-200
-250
-300
-350
(ppm)

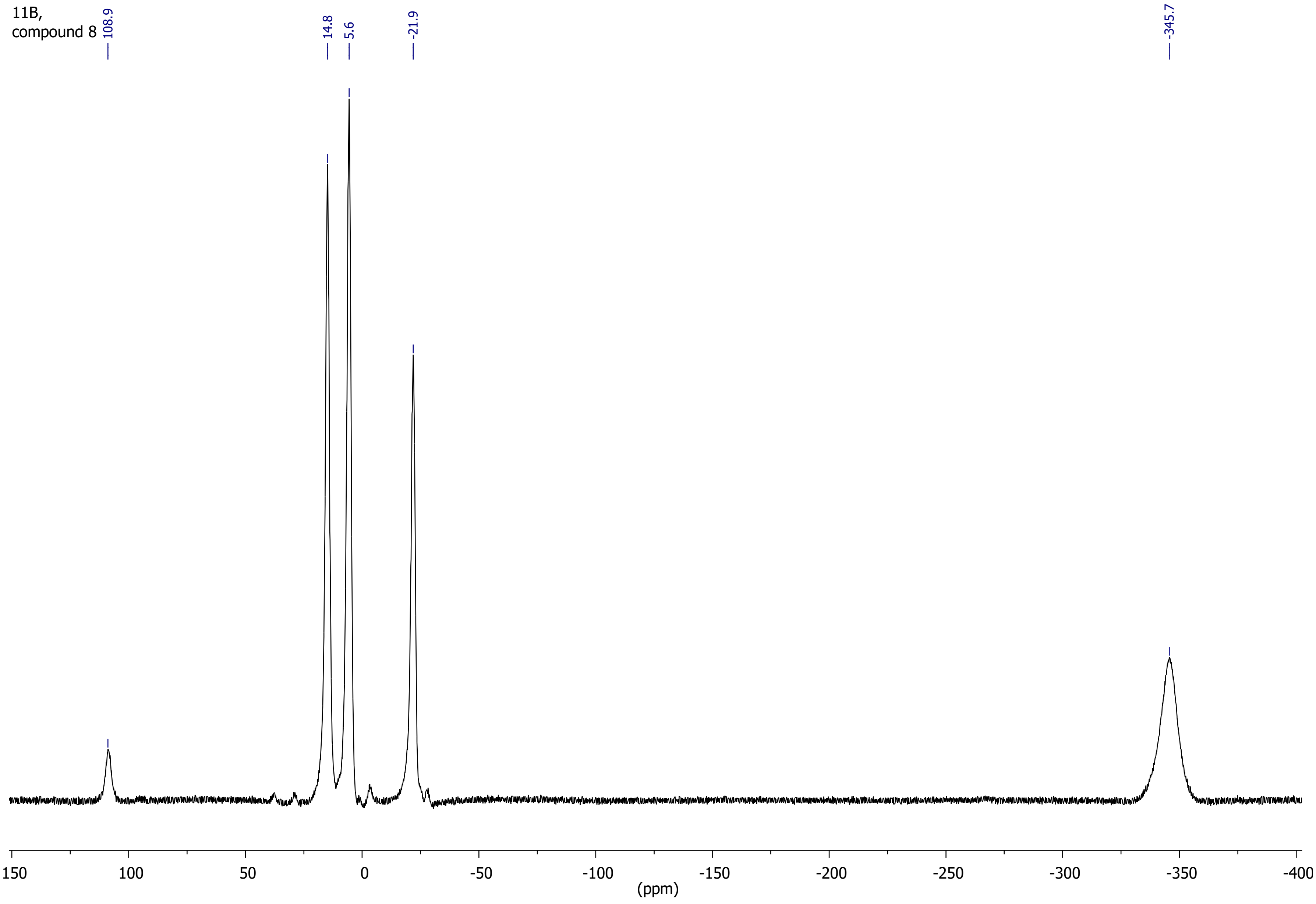
11B,
compound 8
108.9
14.8
5.6
-21.9
-345.7
150
100
50
0
-50
-100
-150
-200
-250
-300
-350
-400
(ppm)

13C{1H}, compound 8

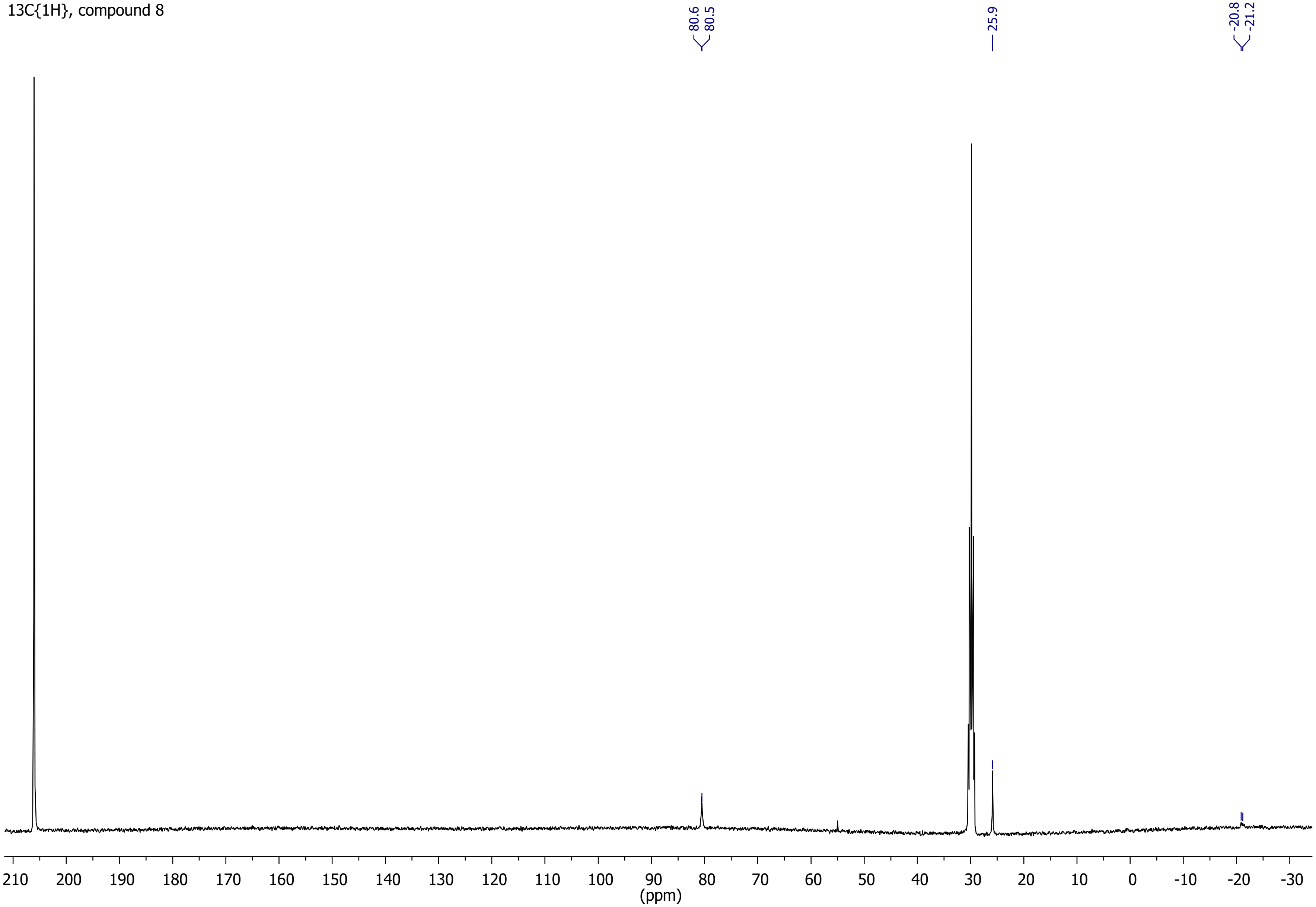

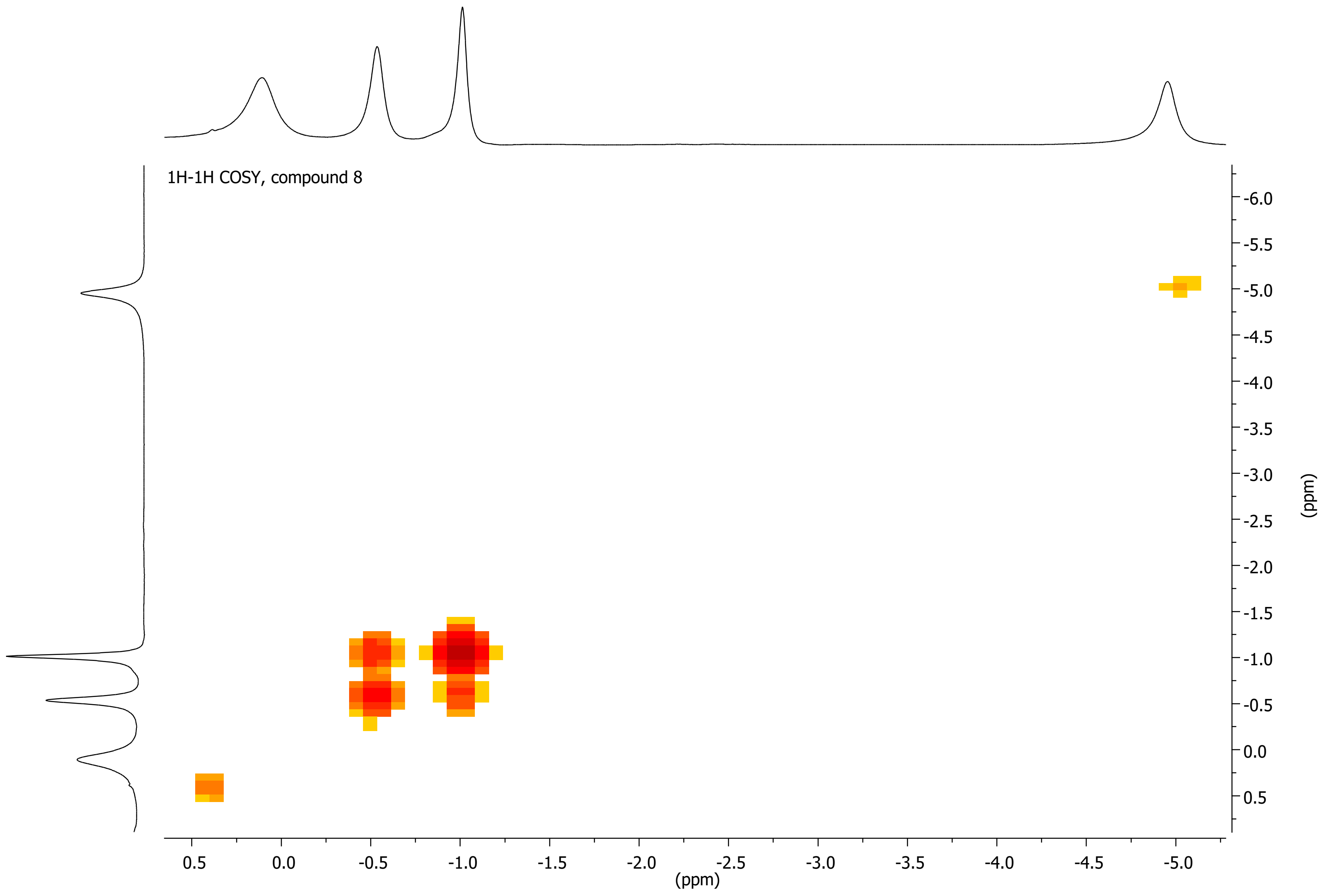
1H-1H COSY, compound 8
0.5
0.0
-0.5
-1.0
-1.5
-2.0
-2.5
-3.0
-3.5
-4.0
-4.5
-5.0
(ppm)
-6.0
-5.5
-5.0
-4.5
-4.0
-3.5
-3.0
-2.5
-2.0
-1.5
-1.0
-0.5
0.0
0.5
(ppm)

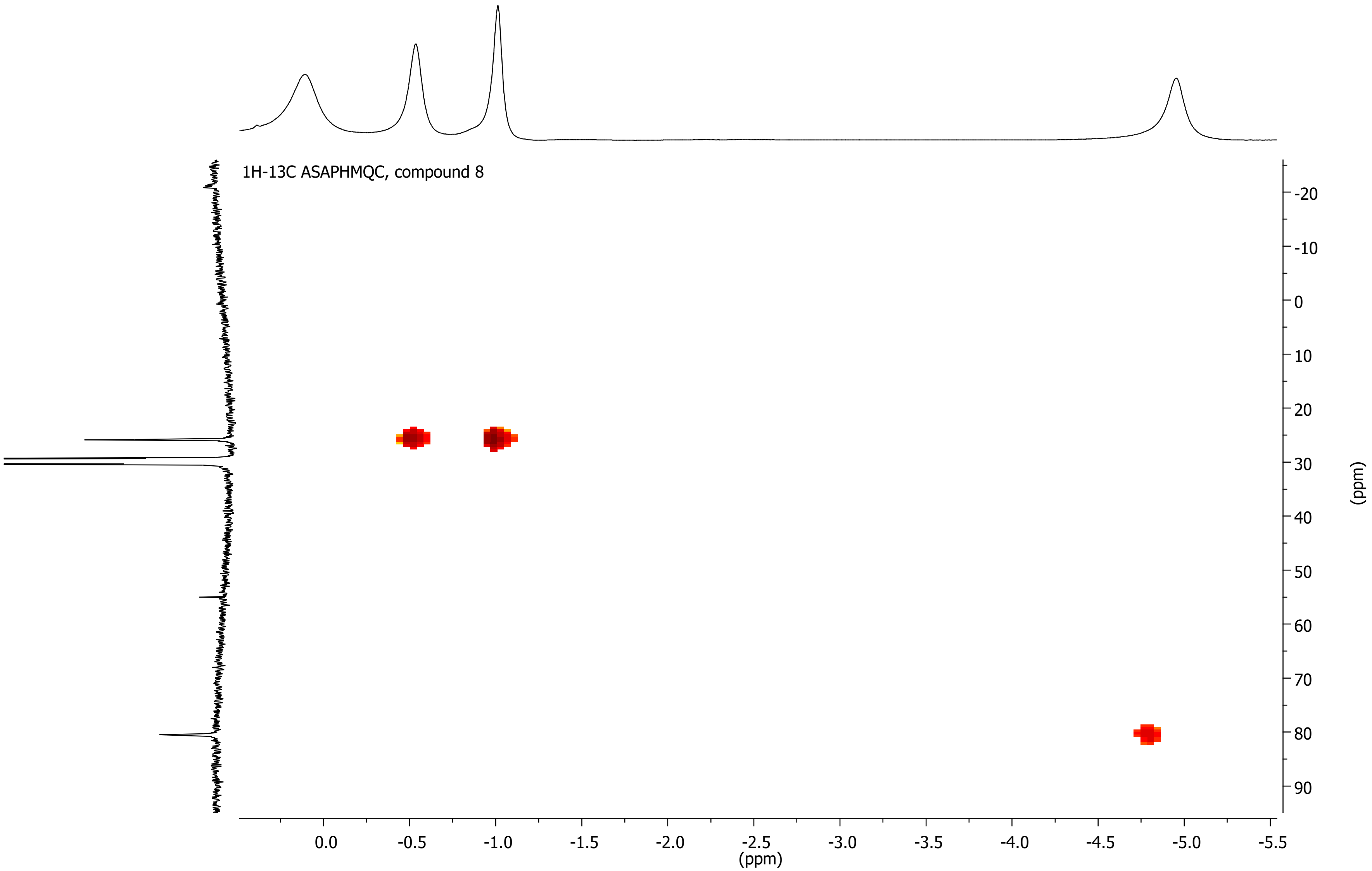
1H-13C ASAPHMQC, compound 8
-20
-10
0
10
20
30
40
50
60
70
80
90
(ppm)
0.0
-0.5
-1.0
-1.5
-2.0
-2.5
-3.0
-3.5
-4.0
-4.5
-5.0
-5.5
(ppm)
